# Disentangling the complexity of tropical small-scale fisheries dynamics using supervised Self-Organizing Maps

**DOI:** 10.1371/journal.pone.0196991

**Published:** 2018-05-21

**Authors:** Manuel Mendoza-Carranza, Elisabet Ejarque, Leopold A. J. Nagelkerke

**Affiliations:** 1 El Colegio de la Frontera Sur-ECOSUR. Departamento de Ciencias de la Sustentabilidad, Villahermosa, Tabasco, México; 2 Inter-University Research Center for Aquatic Ecosystems Research, WasserCluster Lunz–Biologische Station, Lunz am See, Austria; 3 Aquaculture and Fisheries Group, Wageningen University, Wageningen, The Netherlands; Universita degli Studi di Bari Aldo Moro, ITALY

## Abstract

Tropical small-scale fisheries are typical for providing complex multivariate data, due to their diversity in fishing techniques and highly diverse species composition. In this paper we used for the first time a supervised Self-Organizing Map (xyf-SOM), to recognize and understand the internal heterogeneity of a tropical marine small-scale fishery, using as model the fishery fleet of San Pedro port, Tabasco, Mexico. We used multivariate data from commercial logbooks, including the following four factors: fish species (47), gear types (bottom longline, vertical line+shark longline and vertical line), season (cold, warm), and inter-annual variation (2007–2012). The size of the xyf-SOM, a fundamental characteristic to improve its predictive quality, was optimized for the minimum distance between objects and the maximum prediction rate. The xyf-SOM successfully classified individual fishing trips in relation to the four factors included in the model. Prediction percentages were high (80–100%) for bottom longline and vertical line + shark longline, but lower prediction values were obtained for vertical line (51–74%) fishery. A confusion matrix indicated that classification errors occurred within the same fishing gear. Prediction rates were validated by generating confidence interval using bootstrap. The xyf-SOM showed that not all the fishing trips were targeting the most abundant species and the catch rates were not symmetrically distributed around the mean. Also, the species composition is not homogeneous among fishing trips. Despite the complexity of the data, the xyf-SOM proved to be an excellent tool to identify trends in complex scenarios, emphasizing the diverse and complex patterns that characterize tropical small scale-fishery fleets.

## Introduction

In order to understand how complex patterns such as catch rates and species composition of multi-species and multi-gear small-scale tropical fisheries are related to environmental changes through time, it is essential to acquire knowledge on internal fleet dynamics [1–3]. One approach has been the use of multivariate statistics to discover patterns and trends [4, 5], However, some limitations have been observed in the application of classical multivariate methods (e.g., PCA, CCA) or linear based ones (e.g. GLM, GAM), due to the non-linear relationships among variables and the strong interactions between species, fisheries tactics, and target species [6]. In addition, these methods are highly sensitive to extreme data points, large quantities of zero values and cannot handle very large datasets, all of which are common issues in this kind of multivariate datasets.

To understand the complexity of natural patterns in tropical small-scale fisheries, it is necessary to take into account the nature and resolution of the environmental data: complex models require high quality data producing broad confidence intervals; by contrast, simple models present the advantage of low data requirement with low statistical error. These, however, can overlook some important processes, and may fail to consider the non-linear nature of the environment-ecosystem relationship [6, 7, 8].

Many fishery models are based on accurate data sources, especially when derived large and medium-sized fleets with Vessel Monitoring Systems in developed countries [9, 10]. However, the lack of accurate and complete data is common in small-scale multispecies fisheries in developing countries [11, 12]. Fortunately, a valuable source of fishery data is the non-official logbooks made by the owners and retailers of fish reception centers along the coast [13, 14]. Logbook data have been a low-cost but important source of information (e.g., quantities of fish landing, fishing effort, fishing strategies) for mathematical and statistical models [15, 16], taking into consideration mistakes and outliers that are common in this type of data source [14, 17]. The advantages of logbook data sets are: a) the low taxonomic level used in logbooks allows an accurate identification of species in the fishing area b) daily fishery records by vessel are the rule, providing high resolution temporal patterns and c) a large number of vessels can be identified and followed through time. Despite the benefits that logbook data provide for the analysis of tropical small-scale fisheries, few studies have taken advantage of such information [13, 14]. Hence, in this study we use such data with a neural network analysis in order to further understand fisheries dynamics in relation to catch rates and composition (e.g.[18, 19]).

Because of the limitations of common statistical methods (e.g., non-linearity, high interaction between species, extreme data, zero values) [20], a recently emerging option to deal with complex data are artificial neural networks. Unlike traditional methods, neural networks can deal with large heterogeneous and multivariate data sets, as those present in logbooks [21, 22, 23]. Previously, artificial neural networks have been used to explore the non-linear nature of catch-per-unit-effort (CPUE) and have been found to be less sensitive to outliers [14, 24, 25]. Self-organizing maps (SOM) are a specific type of artificial neural network, characterized by an unsupervised learning process. Therefore, previous knowledge about the samples and environmental conditions are not needed in the model training [26, 27]. SOM has allowed for example, to discover complex patterns in catch trends and species abundances in long-term fishery data [23, 28], to model the spatial distribution of fish species in relation to environmental conditions [29], and to generate sustainability indexes for commercial species [30].

The main advantage of SOM is the visualization and synthesis of multidimensional data in few dimensions, usually in a two-dimensional grid composed by nodes [4, 22, 27]. Despite its advantages and applications in complex problems, issues regarding the relationship between the size of the SOM and its power of prediction remain understudied [14]. Additionally, when data complexity is high, a flexible combination of the SOM approach with supervised learning schemes has been recommended [26, 31]. In this line, the supervised mapping version of SOM (xyf-SOM) has been found to present a higher capacity for classification and to reduce computation time substantially [27, 31, 32].

In this study the small-scale marine fishery at the Tabasco coast in Mexico, is used as a model of a situation with a complex multivariate dataset composed of thousands of data entries of a multispecies fishery with high variability in their catch values. During 2011, 8986 registered vessels of the fleet caught 37 998 t of fish, the eighth largest production at the national level [33]. Despite the fact that Tabasco has the shortest coastline (110 km long, 3.3% of the total coastline of the Mexican Gulf and Caribbean Sea), it sustains the activities of 21 499 seashore fishermen (20. 7% of all marine fishermen reported for the Mexican Gulf and Caribbean coasts of Mexico), second only to Veracruz (32 277 fishermen on a 684 km coastline) [34]. The most important marine small-scale fleet of Tabasco is located at the San Pedro port in the municipality of Centla. It presents a high heterogeneity in catch rates [35]; thereby, providing an ideal scenario to study the dynamics and behavior of tropical marine small-scale fisheries. The objective of this study was to develop a xyf-SOM model to characterize the main patterns of catches of a small-scale fishery fleet in relation to the exploited species, and to analyze the variability of catch rate patterns of main species through time (i.e., seasons and years).

## Materials and methods

### Study area and fleet characteristics

**Fig 1:**
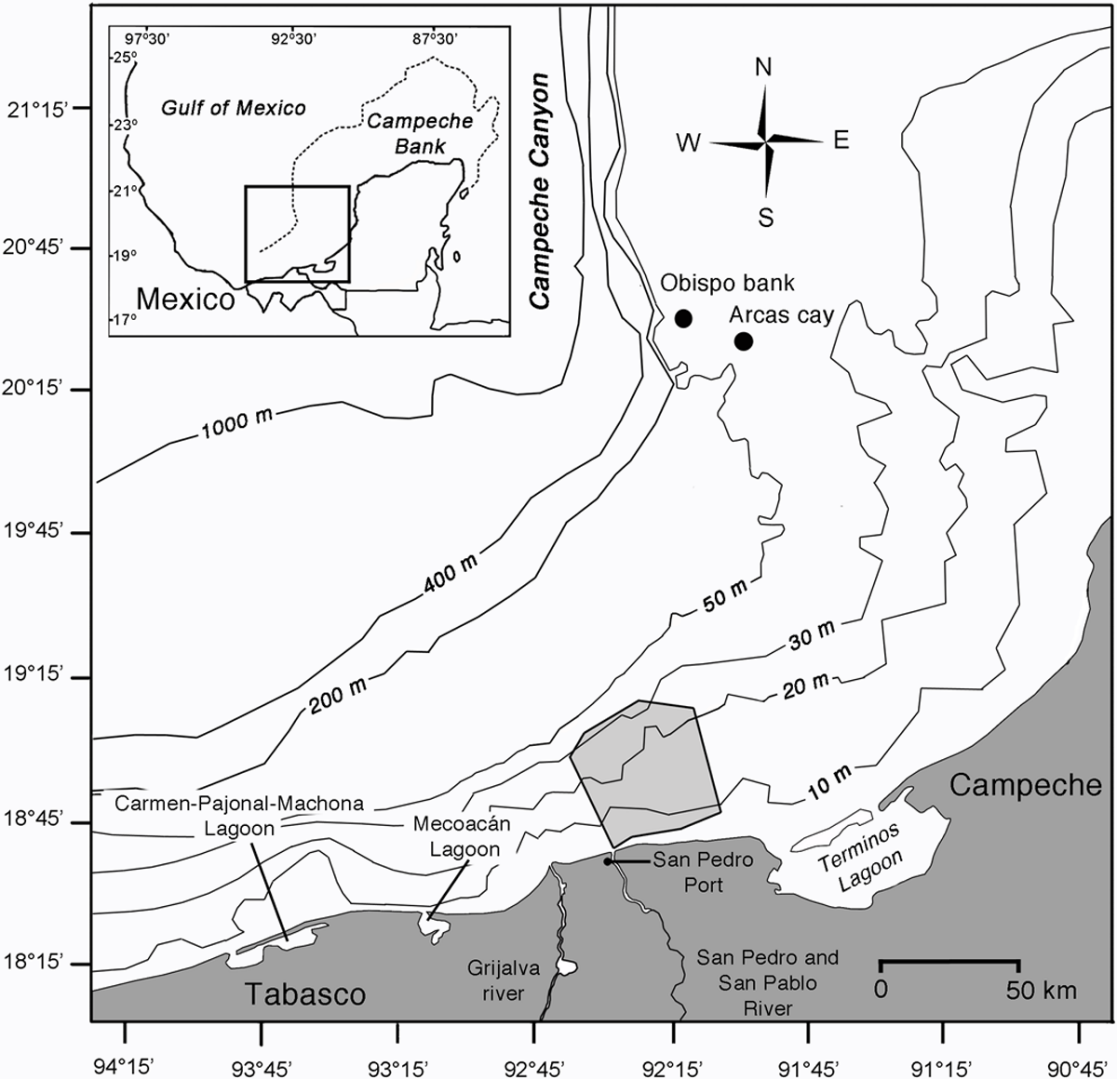
Study area. The light-grey polygon indicates the fishing area of the small-scale fleet from San Pedro Port in the southern Gulf of Mexico.

The fishing area of the San Pedro port small-scale fleet is located on the Campeche Bank (18°40'38", 19°05'25" N and 92°27'07", 92°05'11" W) covering an area of 532 km^2^ (Fig 1). The fishing area is limited by the ca. 5m isobath in the south reaching a maximum depth of 40–50 m to the north at ca. 50 km offshore. The western limit is the Grijalva-Usumacinta river plume, and the eastern limit is an imaginary line starting in the front of the El Carmen mouth of the Términos Lagoon towards the northwest (Fig 1). In addition, the coral reef communities of the Arcas Cay and the Obispo Bank are important areas for the snapper fishery [36]. High levels of productivity characterize the southern Campeche Bank. This productivity is sustained by the freshwater discharge of the Grijalva-Usumacinta river basin, the second largest in the Gulf of Mexico [37]. In addition, extensive nursery areas of economically important fishes surround this area, including the Términos, Mecoacán and Carmen-Pajonal-Machona lagoons and the Grijalva-Usumacinta River delta at the Centla Wetland Biosphere Reserve [38]. Based on sea surface temperature from satellite imagery [39], two seasons were defined: a cold season (< 28°C) from November to April and a warm season (> 28°C) from May to October. However, sea surface temperature is highly dynamic and influenced by surface currents and a mild upwelling in front of the Tabasco coast with its maximum development during July and August [40]. Despite these dynamics, our determined seasons closely correspond to found in climatic and oceanographic analyses covering eight-year of data at the same area [40, 41]. Another important factor affecting our study area is the seasonal drainage regime of the Grijalva-Usumacinta basin on Gulf of Mexico. Discharge is highest during September to November, while it is lowest during April [42].

Based on the fishing gears and target species, the San Pedro port fishing fleet can be divided in two groups of fishermen. The first group consists of “one-day fishermen”, who use artisanal bottom longlines (BL) without specific target species. Around 98% of the species caught have some commercial value [35, 43]. The crew of a fishing trip is typically composed of two fishermen and its duration is limited to one day (12–14 hours). The second group consist of “three-day fishermen”, who use vertical lines (VL) with 10 to 15 hooks, locally named “ristra” [44]. Three to four fishermen are involved in a typical trip, using an equal number of vertical lines. Contrary to the first group, these fishermen have one target species, the red snapper *Lutjanus campechanus* one of the most valuable fish species in the Gulf of Mexico [45, 46]. Nevertheless, this is also a multispecies fishery and fishermen sell all other species caught (mainly a diversity of snappers and groupers of the genera *Lutjanus*, *Ephinephelus* and *Hyporthodus*). In addition to the vertical line, some fishermen use a bottom longline specific for shark capture (SBL), locally named “cimbra” [47], with approximately 100 hooks. Since SBL is additional to the VL, the fishing time is the same (3–4 days), the two gears are operated independently: VL during the day and SBL at night. Other gears were not included in the analysis since they occur in low frequencies (e.g. gillnets occur in <5% of the total number of fishing trips). The vessel characteristics of both fleets are very homogeneous over time; all are fiberglass vessels of 7.5 m long, with a 300–350 kg storage capacity and a 60–120 hp outboard engine [35].

### Dataset

**Fig 2:**
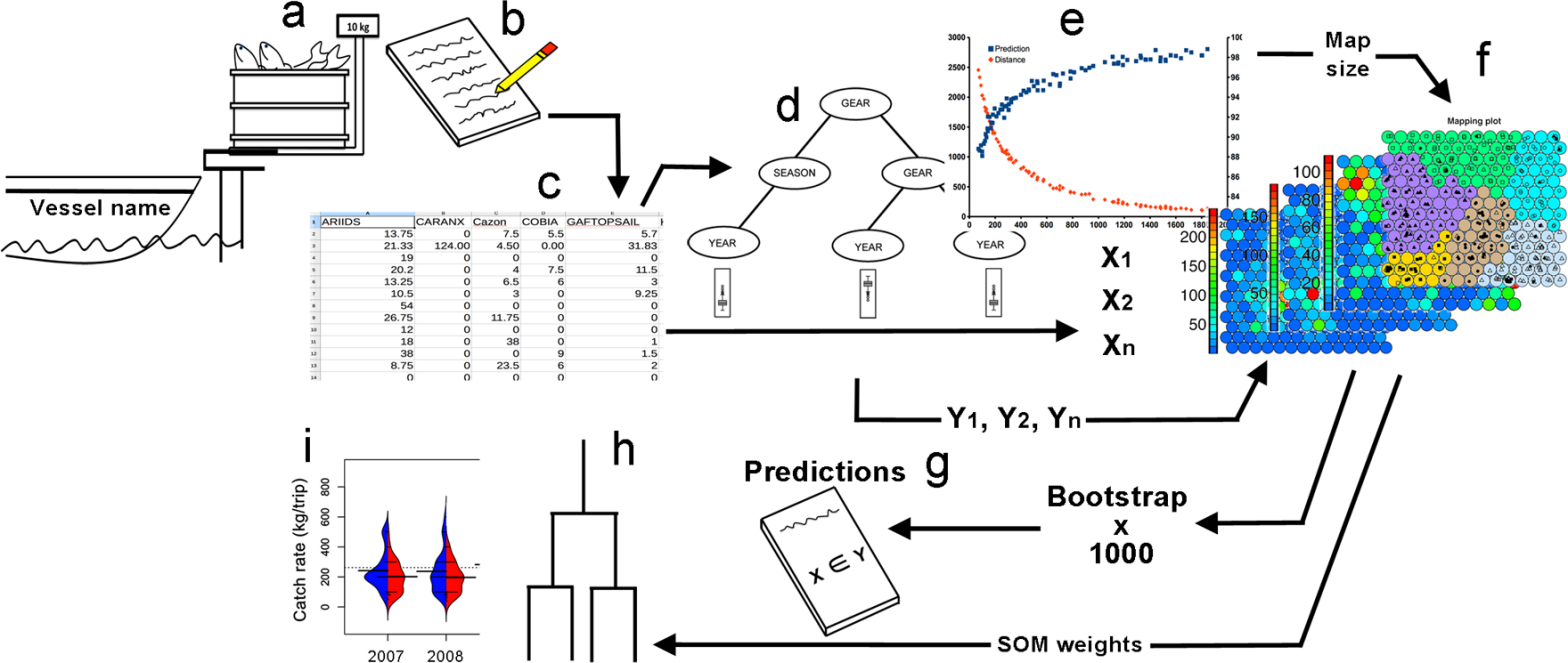
Graphical summary of the methodology. a) Vessel arrival identified by fishermen nickname of vessel name, b) data acquisition in logbook, c) ordering and classifying data in electronic format, d) defining the importance of factor by ctree methodology, e) definition the size of xyf-SOM grid, f) xyf-SOM training, g) generation of confidence intervals for xfy-SOM predictions, h) testing for species arrangement based on xyf-SOM weights using cluster analysis, i) catch rate analyses using beanplots on xyf-SOM weights.

The dataset was obtained from logbooks containing daily individual entries by trip (Fig 2A). Each trip was identified by the name or nickname of the vessel and the fisherman responsible for the trip. Based on these names and interviews, we identified the fishing gear used in each fishing trip. The logbook contains a comprehensive species list (common names). An employee of the fish trading company, with experience in fish identification, weighed the fish by species or group of species (e.g. sharks) and filled in the logbooks (Fig 2B). Validation between the common names of the logbooks and scientific names was performed during monthly field sampling surveys in 2006–2012, by interviews supported by photographs from Hoese and Moore [48], Reséndez [49] and Carpenter [50]. Weights were recalculated as eviscerate weight. Sharks were not identified to the species level but classified into three commercial groups: a) “tripa” (sharks <2kg), b) “cazon” (sharks from 2 to 20 kg), and c) “tiburon” (sharks >20 kg).

The primary dataset covers the period from November 2006 to October 2012; containing a total of 7120 fishing trips (5534 for BL, 820 for VL and 765 for VL + SBL), with 23662 records of species weights. However, no precise spatial reference for fishing trips is available, having only a general fishery area (Fig 1).

### Data analysis

For all the analyses, fish were grouped into 22 groups based on their relative importance in the catches and their taxonomic affinities, fish common names were based on Nelson et al. [51] (Table 1 and Fig 2C). Except barracuda, largehead hairtail, sand weakfish, tripletail, bearded brotula little tunny, snook and “Other fishes” (<0.5% of total catch volume), all other groups were used in the subsequent analyses. Despite the general differences in trip duration between the two fleets (one day for BL and two or three days for VL+SBL and VL), the information on the duration of individual trips was imprecise and all analyses were based on the catch rates by trip, expressed in kg/trip [5, 52].

**Table 1:** Fish groups and species caught by the small-scale fleet of San Pedro port in Tabasco, Mexico. Total weight percentage and catch rates (kg/ day) are presented by species and gear combination.

Fish category	Order	Family	Species	Common name	Total weight (%)				Catch rate (kg / trip day)		
					Total	Bottom longline	Vertical line	Vertical line + shark line	Bottom longline	Vertical line	Vertical line + shark line
Sharks	"Sharks"	"Sharks"	Tiburon (ind.> 25 kg)	Tiburon	1.7	0.2	<0.1	9.7	26.7 ± 27.5	17.7 ± 18.7	98.5 ± 90.2
			Cazon (ind. 2–25 kg)	Cazon	4.4	3.3	0.1	12.6	20.0 ± 44.1	4.1 ± 3.1	45.7 ± 66.0
			Tripa (ind. < 2 kg)	Tripa	1.1	1.2	<0.1	1.5	11.4 ± 23.1	4.8 ± 4.6	34.5 ± 62.7
Rays	Myliobatiformes	Myliobatidae	*Aetobatus narinari*	Spotted eagle ray	0.8	1.1	<0.1	<0.1	23.1 ± 2.3	6.0	13.1 ± 7.9
		Dasyatidae	*Dasyatis americana*	Southern stingray	18.4	23.4	0.1	10.2	57.6 ± 68.7	7.1 ± 7.6	58.9 ± 61.5
		Gymnuridae	*Gymnura micrura*	Smooth butterfly ray	0.8	1.1	<0.1	0.1	12.8 ± 25.2	6.2 ± 2.9	13.5 ± 28.1
Tarpon	Elopiformes	Megalopidae	*Megalops atlanticus*	Tarpon	0.6	0.9	<0.1	<0.1	5.3 ± 43.0	2.5	13.6 ± 16.0
Gafftopsail sea catfish	Siluriformes	Ariidae	*Bagre marinus*	Gafftopsail catfish	34.9	48.6	0.2	0.1	46.3 ± 63.4	5.9 ± 4.8	14.3 ± 42.1
Hardhead sea catfish			*Ariopsis felis*	Hardhead catfish	6.1	8.6			23.2 ± 23.8		
Bearded brotula	Ophidiiformes	Ophidiidae	*Brotula barbata*	Bearded brotula	0.1	<0.1	0.3	<0.1	6.7 ± 6.0	7.5 ± 7.6	8.4 ± 11.6
Amberjacks	Perciformes	Carangidae	*Seriola dumerili*	Greater amberjack	0.7	0.1	1.7	2.3	15.7 ± 21.0	15.3 ± 2.5	2.7 ± 22.4
			*Seriola rivoliana*	Almaco jack	<0.1	<0.1	0.1	0.1	17.0 ± 8.7	12.1 ± 1.4	25.9 ± 46.2
			*Seriola zonata*	Banded rudderfish	0.4	<0.1	0.9	1.3	19.9 ± 29.5	16.7 ± 23.2	27.9 ± 36.1
Jacks	Perciformes	Carangidae	*Caranx crysos*	Blue runner	<0.1	<0.1	<0.1	<0.1	33.6 ± 26.4	21.0	1.5 ± 3.9
			*Caranx hippos*	Crevalle jack	0.8	1.0	0.1	0.2	38.1 ± 55.8	33.9 ± 63.3	16.7 ± 1.4
			*Caranx latus*	Horse-eye jack	0.1	0.1	0.1	0.3	3.6 ± 28.1	21.0 ± 26.0	31.2 ± 62.4
			*Caranx ruber*	Bar jack	<0.1	<0.1			6.6 ± 8.1		
Pompano	Perciformes	Carangidae	*Trachinotus carolinus*	Florida pompano	3.2	4.4	<0.1	0.1	42.5 ± 47.5	21.1 ± 28.1	84.2 ± 63.5
Snooks	Perciformes	Centropomidae	*Centropomus paralellus*	Smallscale fat snook	<0.1	<0.1			6.3 ± 8.0		
			*Centropomus undecimalis*	Common snook	0.1	0.2	<0.1	<0.1	9.8 ± 1.2	8.0 ± 8.3	19.1 ± 5.4
Tripletail	Perciformes	Lobotidae	*Lobotes surinamensis*	Atlanctic tripletail	<0.1	0.1	0.1	<0.1	8.7 ± 13.6	35.6 ± 29.0	2.7 ± 0.3
Snappers	Perciformes	Lutjanidae	*Lutjanus analis*	Mutton snapper	0.5	<0.1	1.1	2.1	11.8 ± 14.1	23.8 ± 32.6	27.5 ± 43.6
			*Lutjanus buccanella*	Blackfin snapper	0.1		0.2	0.2		7.7 ± 9.7	11.2 ± 22.3
			*Lutjanus campechanus*	Red snapper	14.8	3.2	52.1	35.0	37.7 ± 46.2	61.9 ± 57.8	57.0 ± 59.2
			*Lutjanus cyanopterus*	Cubera snapper	<0.1	<0.1	0.1	0.1	53.0 ± 93.8	17.3 ± 1.3	23.1 ± 13.8
			*Lutjanus griseus*	Grey snapper	0.2	0.1	0.6	0.4	12.4 ± 16.5	14.6 ± 22.6	9.3 ± 11.2
			*Lutjanus jocu*	Dog snapper	0.2	<0.1	0.4	0.7	9.0 ± 9.5	16.0 ± 18.5	18.7 ± 26.2
			*Lutjanus synagris*	Lane snapper	1.9	0.2	9.1	3.6	1.8 ± 17.3	19.8 ± 27.8	14.7 ± 22.2
			*Ocyurus chrysourus*	Yellowtail snapper	<0.1	<0.1	<0.1	<0.1	2 ± 1.4	8 ± 8.6	13.9 ± 22.1
Vermilion snapper	Perciformes	Lutjanidae	*Rhomboplites aurorubens*	Vermilion snapper	5.0	<0.1	25.9	9.9	13.4 ± 14.5	39.8 ± 45.8	32.4 ± 4.5
Cobia	Perciformes	Rachycentridae	*Rachycentron canadum*	Cobia	0.6	0.9	0.1	0.1	15.6 ± 23.1	15.4 ± 12.3	12.8 ± 8.2
Sand weakfish	Perciformes	Sciaenidae	*Cynoscion arenarius*	Sand seatrout	<0.1	0.1	<0.1	<0.1	9.7 ± 5.7	2.7 ± 1.0	3.7 ± 2.4
Mackerels	Perciformes	Scombridae	*Scomberomorus cavalla*	King mackerel	2.5	1.0	5.0	7.2	71.2 ± 135.2	15.2 ± 145.2	159.7 ± 152.1
			*Scomberomorus maculatus*	Atlantic Spanish mackerel	<0.1	<0.1			52.5 ± 48.0		
Little tunny	Perciformes	Scombridae	*Euthynnus alletteratus*	Little tunny	0.2	0.3	<0.1		259.4 ± 439.9	33.0	
Tunas	Perciformes	Scombridae	*Thunnus atlanticus*	Blackfin tuna	<0.1	<0.1	<0.1	0.1	1.0	6.0 ± 2.8	12.0 ± 6.1
			*Thunnus albacares*	Yellowfin tuna	0.1	<0.1	0.2	0.3	14.9 ± 15.5	15.7 ± 21.9	21.1 ± 42.3
Groupers	Perciformes	Serranidae	*Epinephelus adscensionis*	Rock hind	<0.1	<0.1	<0.1	0.1	2.2 ± 0.3	4.8 ± 6.5	14.3 ± 24.9
			*Epinephelus morio*	Red grouper	0.0	<0.1	0.1	0.1	17.5 ± 9.1	9.3 ± 14.1	25.9 ± 45.2
			*Hyporthodus nigritus*	Warsaw grouper	0.1	<0.1	0.4	0.3	43.0	2.4 ± 3.4	19.4 ± 4.0
			*Mycteroperca interstitialis*	Yellowmouth grouper	0.1	<0.1	0.6	0.4	1.5 ± 0.7	24.3 ± 4.7	18.3 ± 23.0
Barracuda	Perciformes	Sphyraenidae	*Sphyraena barracuda*	Great barracuda	0.1	<0.1	0.1	<0.1	14.6 ± 1.4	18.1 ± 22.4	12.5 ± 1.5
Largehead hairtail	Perciformes	Trichiuridae	*Trichiurus lepturus*	Atlantic cutlassfish	<0.1	<0.1	<0.1	<0.1	16.1 ± 12.1	8.7 ± 8.8	1.7 ± 0.3
Other fishes	Anguilliformes	Ophichthidae	*Ophichthus rex*	King snake eel	0.1	<0.1	0.2	0.2	6.2 ± 4.2	17.4 ± 35.2	8.9 ± 15.5
	Aulopiformes	Synodontidae	*Synodus foetens*	Inshore lizardfish	<0.1	<0.1		<0.1	8.6 ± 7.1		23.0
	Tetraodontiformes	Tetraodontidae	*Lagocephalus laevigatus*	Smooth puffer	<0.1	<0.1			4.5 ± 55.8		
	Perciformes	Coryphaenidae	*Coryphaena hippurus*	Dolphinfish	0.1	0.1	0.1	0.1	16.8 ± 39.5	12.3 ± 18.8	8.1 ± 5.6
		Ephippidae	*Chaetodipterus faber*	Atlantic spadefish	<0.1	<0.1	<0.1		12.5 ± 14.3	7.2 ± 6.7	
		Sciaenidae	*Micropogonias undulatus*	Atlantic croaker	<0.1	<0.1	<0.1	<0.1	3.2 ± 1.7	1.0	2.0 ± 0.5
		Sparidae	*Calamus leucosteus*	Whitebone porgy	<0.1	<0.1	<0.1	<0.1	4.1 ± 3.0	1.8 ± 1.2	7.2 ± 8.6

#### Analysis of factors affecting catch rates: Confidence inference trees

We constructed a conditional inference tree to identify the degree of importance of factors affecting catch rates, using the ctree routine in the package ‘party’ from R software [53, 54]. The factors included in the analysis were year (2007–2012), season (cold and warm), and gear type (bottom longline, vertical line, and shark bottom longline; Fig 2D). This method uses non-parametric methods as splitting criteria, based on multiple comparison tests, thereby resulting in an unbiased predictor selection. The criteria to stop the tree is also based on a multiple test procedure [54]. Because the confidence inference tree method uses non-parametric methods, it can support all kinds of data types, including nominal, ordinal, numeric, and multivariate response variables [53].

#### Internal and general patterns of fleets: Supervised self-organizing map (xyf-SOM)

We used self-organizing maps (SOM), a type of neural network, to analyze the relationships and variability between individual fishing trips within the fleets, species catch rates and inter-annual and seasonal variability [55, 56]. Self-Organizing Maps has been applied to fishery problems related to the analysis of logbook fishery data in tuna fishery [24], tropical small scale fisheries [28] and the heterogeneity of fishing practices [14]. A SOM consists of a grid composed of units named nodes or neurons, in which nodes that are ordered by similarity among neighbors. Each node is defined by a weight vector (or codebook vector) with the same dimensions as the dataset [27, 28]. In our case the weight vector of each node consists of the catch rates of each fish category. A standard SOM algorithm starts with initial weight vectors for each node, resulting in a ‘prototype’ pattern. Usually this prototype pattern is made by randomly assigning a subset of the data to the nodes. During the following training process to construct the SOM, objects (in our case fishing trips) are repeatedly presented in random order to the nodes of the map. The weight vector of the node which is most similar to the current training object (the “winning” node) will be updated to become even more similar [27, 57]. Updating is done by weighted averaging of the node and the training object, with the training object usually having a small weight, known as the learning rate α. This rate decreases during training in order for the map to converge. Not only the “winning” node, but also the nodes in the immediate neighborhood will be updated, since close neighbors need to be similar in SOM. During training the size of the neighborhood will decrease, so eventually only the “winning’ nodes will be adapted [57]. Training will be performed in a preset number of iterations.

The SOM algorithm, as described in the previous section, is based on “unsupervised” exploratory analysis. However, since we are interested in how catch rates of the different species are related to season, years, gear types, and individual fishing trips, we used “supervised” mapping a more powerful modeling alternative in situation with complex data [31, 57].

The X matrix of the xyf-SOM is the catch rate in kg/trip for each fish category and the Y matrix contains classification information such as individual fishing trip, gear type, season and year. This capacity to incorporate classification information is the principal attribute of xyf-SOM [31, 57]. The X and Y matrices are concatenated and the xyf-SOM is trained using the similarities in both X and Y space. The result of the training is two concatenated maps: one with the X-variables and other with the Y-variables, but the topology of the nodes is the same in both (they can be projected on top of each other). Since Y variable is a class matrix, containing classification information such as individual fishing trip, gear type, season and year, we employed the Tanimoto distance, which has been reported to produce better classification results [57]. The distances in the spaces of X and Y are calculated separately and both are scaled to the maximum distance (1), so that the overall distance is a weighted sum of both:

D(o,u)=αDx(o,u)+(1−α)Dy(o,u) 1

where D(o,u) is the combined distance of an object o to unit u, and Dx and Dy indicate the distances in the individual spaces. The weights (α) for each the X and Y spaces are selected by the user, in our case we selected α = 0.5 leading to equal weights for the X and Y spaces [57].

The number of iterations for the xyf-SOM training process (rlen) was 1000 and the learning rate α started from 0.05 and linearly decreased to 0.01. First, xyf-SOM produces a set of objects selected randomly from the original dataset. Next, the Y-values for a new object can be predicted given its X-matrix values, which can then be compared to the actual Y-values (using the ‘trainY’ argument) [57].

Because the xyf-SOM grid size is related to the power of prediction [58]; we tested several sizes, looking for the map size with the higher prediction rate and minimum distance (topographic error) between elements, but taking into account the relation between resolution and accuracy [23, 26, 59] (Fig 2E). The starting size of the xyf-SOM grid was based on the formula c = 5√n, where c is the number of nodes and n is the number of samples [23, 60, 61]. After we found the best size of the xyf-SOM (Fig 2F), the mean and the 90% confidence interval of correct classification percentages (prediction power) were estimated using 1000 bootstrap replicas from the new object sets [62] (Fig 2G). Computations were implemented using the ‘prediction.kohonen’ function from the Kohonen package for R software [57].

We applied xyf-SOM analysis for two situations: 1) a general xyf-SOM exploring relations between fishing gears types (BL, SBL, VL), years, seasons (cold, warm) as classifying variables (Y matrix) and catch rates of fish groups by fishing trip (X matrix); and 2) we constructed specific xyf-SOMs for each type of fishing gear (BL, SBL+VL and VL) using the same classifying variables for Y matrix as the general xyf-SOM. The latter allowed for comparison of the prediction power of the maps for isolated gears against the general xyf-SOM. Mapping-procedures were performed using the Kohonen package for R software [57].

A hierarchical cluster analysis based on Ward’s linkage method was performed on the weight vectors produced by the general xyf-SOM (Fig 2H), with the objective to analyze how the xyf-SOM identify relationships between the fish categories [28, 60].

#### Catch rate trends analysis by gear over years and seasons: Beanplots

The weight vectors of the xyf-SOM for the BL, VL and the VL + SBL fleets were used to explore the effects and trends of annual and seasonal patterns on the catch rate (Fig 2I). The graphical analysis was based on beanplots, using the R-package “beanplot” [63]. Beanplots provide density shape estimations, showing the distribution of the data in relation to the general catch rate trends, which is useful in this context in order to compare the individual variability among fishing trips. These density shape estimations are based on the Sheather-Jones method, which means the bandwidth of all groups that are to be compared, thereby allowing direct comparison between groups [64].

Inter-annual and seasonal variability in catch rates based on weight vectors were analyzed for the three fish species with the highest catch rates in each gear type, as well as for the overall catch rates. Differences between catch rates of groups were tested using the non-parametric Kruskal-Wallis test for multiple comparisons [65, 66], because residuals were not normally and homogeneously distributed. Bonferroni corrections were made for multiple *post hoc* comparisons [67].

## Results

### Fish species by gear-fleet

The small-scale fleet of San Pedro port exploits ca. 50 fish species, grouped in 27 families and 22 functional groups (Table 1). The three most abundant species by total weight (68.1% of the total catch weight in 2007–2012, Table 1) were gafftopsail catfish *Bagre marinus* (34.9%), the southern stingray *Dasyatis americana* (18.4%) and the red snapper *Lutjanus campechanus* (14.8%). The highest number of species was found in the BL fishery (46 species), followed by the VL (44) and VL+SBL (43) fleets (Table 1).

For the BL fleet, catches of the gafftopsail catfish were most important (48.6% of catch weight), followed by rays (25.6%), and hardhead catfish *Ariopsis felis* (8.6%, Table 1). Highest mean catch rates for BL were found for little tunny *Euthynnus alletteratus*, and king mackerel *Scomberomorus cavalla* with 259.4±439.9 and 71.2±135.2 kg/trip respectively. However, these species were caught in less than 1% of the trips and therefore their importance for total catch weight was low (0.3 and 1.0%, Table 1). The mean catch rates of the most abundant fish species (the gafftopsail catfish, the southern stingray and the hardhead catfish) were 46.3±63.4, 57.6±68.7 and 23.2±23.8 kg/trip respectively, occurring in more than 90% of the catches (Table 1).

For the VL fleet, the most important fish group by total weight was the snappers (63.7%) with red snapper as the most important species (52.1%, Table 1). Second was the vermilion snapper *Rhomboplites aurorubens* (25.9%). The highest catch rates in the VL fleet were also found for red snapper (61.9±57.8 kg/trip) and vermilion snapper (39.8±45.8 kg/trip, Table 1). Both species occurred in more than 95% of the catches.

For the VL+SBL fleet, in addition to snappers and vermilion snapper, sharks contributed most to the total catch weight (23.8%), with cazon being most important (12.6%), followed by tiburon (9.7%, Table 1). southern stingray contributed 10.2% of the total catch weight (Table 1). The highest mean catch rate for the VL+SBL fishery was found for king mackerel (159.7±152.1 kg/trip), although its importance for the total catch weight was low (7.2%), because it only occurred in less than 8% of the catches. tiburon (98.5±90.2 kg/trip), southern stingray (58.9±61.5 kg/trip), red snapper (57.0±59.2 kg/trip), cazon (45.7±66.0 kg/trip) and vermilion snapper (32.4±4.5 kg/trip) all had high catch rates and occurred in 97% of the catches (Table 1).

### Factors affecting catch rates

**Fig 3:**
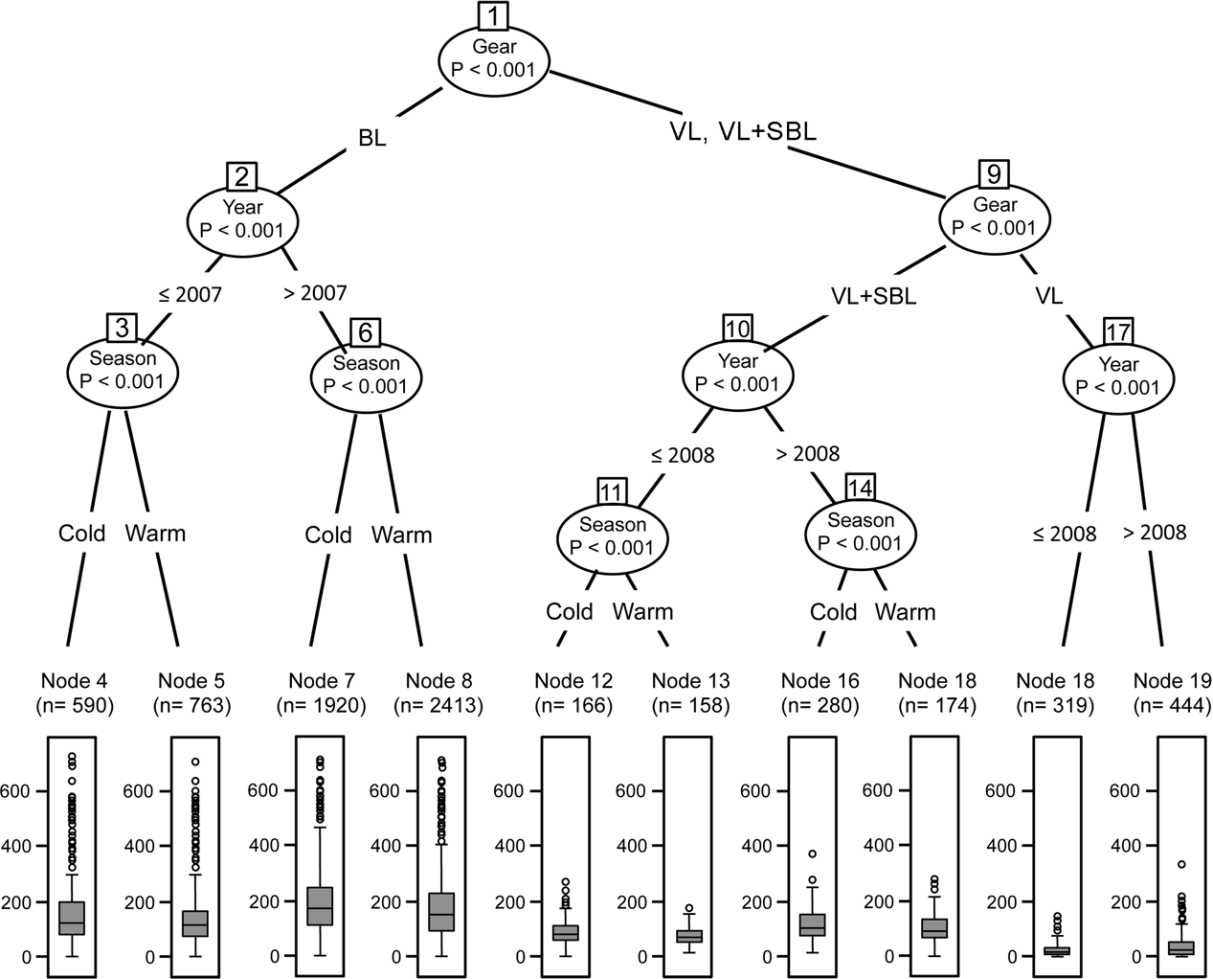
Conditional inference tree. Main factors influencing catch rates of the small-scale fishery fleets of San Pedro port, Tabasco, Mexico. BL = bottom longline, VL = vertical line, SBL = Shark bottom longline. Boxplots below the tree are catch rates (mean±S.D).

The conditional inference tree (Fig 3) indicated that the most important factor influencing overall catch rates is the fishing gear type (nodes 1 and 9). Node 1 separates the catch rates from the one-day fishery with BL on the left branch (high catch rates), from the lower catch rates from the three-day fishery with VL, or with VL+SBL on the right branch (Fig 3). Node 9 further separates the three-day fisheries into a right branch (VL with lowest catch rate), and at the left branch VL+SBL, with higher catch rates in relation to VL (Fig 3).

The second most significant factor influencing the catch rate is the year (nodes 2, 10, and 17). For the BL fleet, the differences between 2007 and the later years 2008–2012 are most important (Fig 3), while for the VL fleet (node 17) and for the VL+SBL fleet (node 10) the main differences in catch rates are between 2007–2008 and 2009–2012 (Fig 3). Finally, at the bottom of the tree, season influenced the catch rates for the BL and the VL fleets, but not for the VL+SBL fleet (Fig 3).

### Determining the optimal size of the xyf–SOM

**Fig 4:**
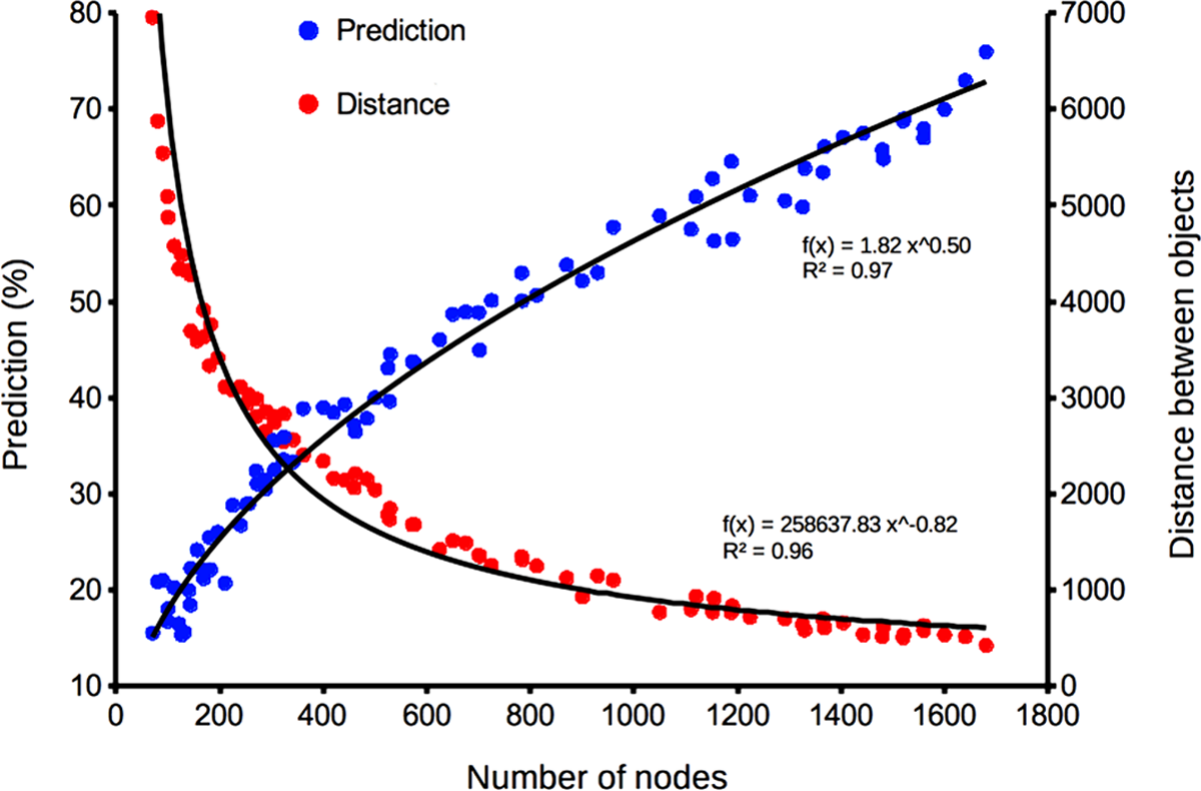
Determination of the xyf-SOM map size. Relationship between number of nodes (xyf-SOM size) and prediction percentage (blue circles), and distance between objects inside the xyf-SOM (red circles).

The prediction power shows a potential relationship with the map size [Prediction power = 1.82 (number of nodes) ^0.50^, r^2^ = 0.92], this relationship is maintained independently of the map form (Fig 4). On the other hand, the distance between objects presented a negative potential relation with the map size [distance = 2.59 (number of nodes) ^-0.82^, r^2^ = 0.96] (Fig 4). Based on these results, we selected the xyf-SOM grid size with the highest power of prediction (40 x 42 nodes).

### Fleets and species patterns, the xyf-SOM

**Fig 5:**
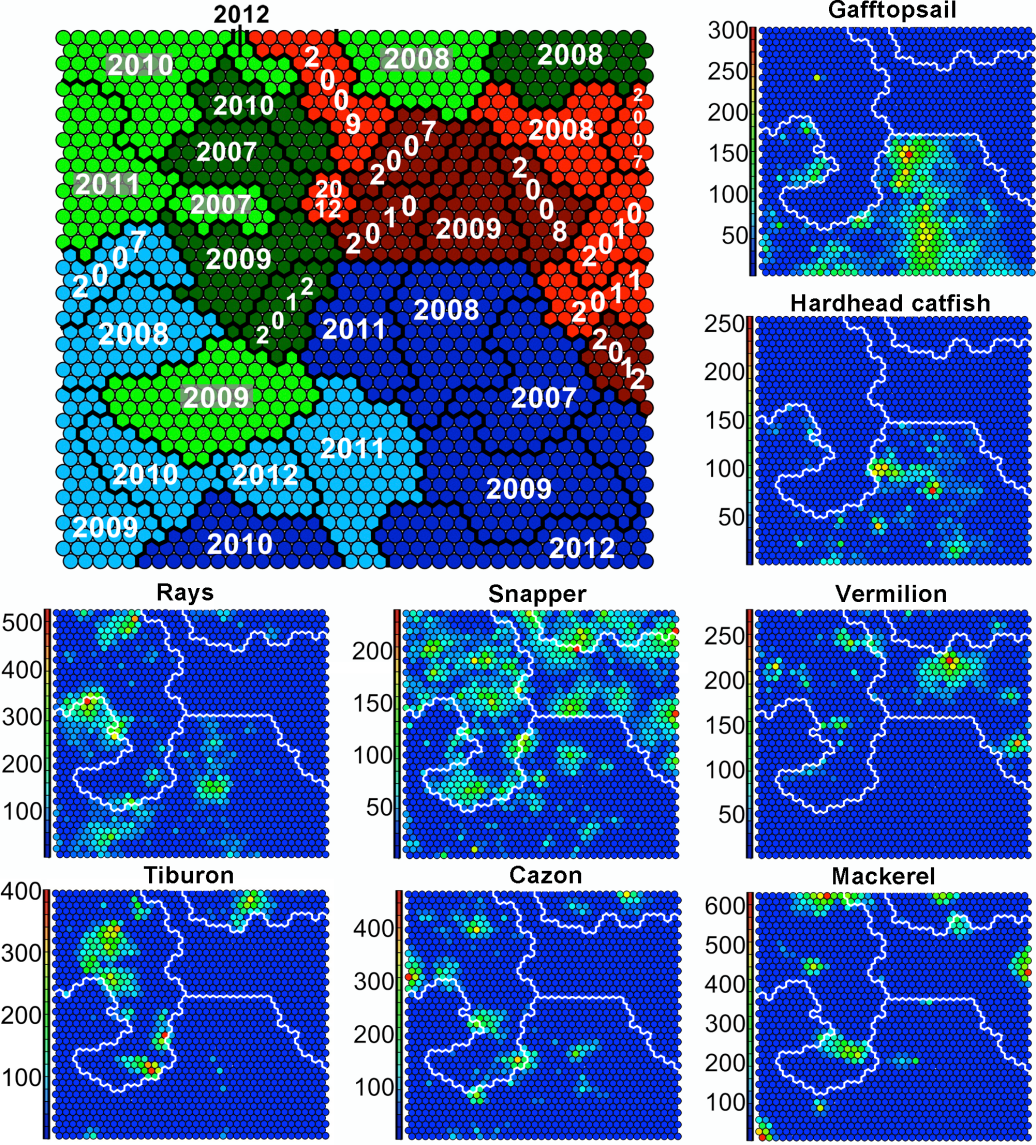
Supervised Self-Organizing Map (xyf-SOM). At the general xyf-SOM map, color indicates gear type: bottom longline (BL) is represented in blue, vertical line + shark bottom longline (SBL) in green and vertical line (VL) in red; light tones indicate the cold season, and dark tones the warm season. Thick lines delineate years. At the species-specific xyf-SOM maps, lines indicate the predicted areas for BL, VL and SBL, whereas the color scale corresponds to the catch rate in kg/trip (blue is low, red is high).

The xyf-SOM classified individual fishing trips based on gear type, season and year (Fig 5). Within the xyf-SOM three predicted areas for each gear type are indicated blue colors = BL, (green colors = SBL + VL, red colors = VL, Fig 5). Further, in each gear area seasons are represented by tone (clear tones = cold season, dark tones = warm season), and lines separate the years areas. Area sizes are related to the number of fishing trips for each factor included (Fig 5).

The gear-season-year configuration is related to the species catch rates inside the SOM, so that it is easy to distinguish the species characterizing each gear type (Fig 5). For instance, gafftopsail catfish catch rates (kg/trip) were associated with the area occupied by the BL fleet (blue color), especially in the warm season (Fig 5). Fishermen using BL that did not acquire high catch rates of gafftopsail catfish can be seen to be more associated with high catch rates of rays or hardhead sea catfish (Fig 5). In case of rays the highest catch rates for BL fleet were related to the cold seasons, especially during 2011 (Fig 5).

Fishermen using the VL and VL+SBL reached the highest catch rates for snappers. As mentioned for BL, different fishermen within fleets using VL and VL+SBL have different target species or they switch between several target species. Low snapper catch rates were usually compensated by higher catch rates of rays, tiburon, vermilion snapper, or mackerels (Fig 5). Elasmobranchs as tiburon (sharks >20 kg) and cazon (sharks from 2 to 20 kg) were related to the VL+SBL fleet areas in xyf-SOM (green color). However, the BL fleet also caught cazon (Fig 5). High catch rates of mackerels were taken by some fishermen with any of the three fishing gears, indicating the non-specificity of mackerels to any gear. However, the limited number of trips in which mackerels were taken indicates that either they occur erratically, or that only some fishermen specifically targeted on this species (Fig 5). It should be noted that not only the more abundant fish groups, shown in the figures, are included in these predictions; but also the other less abundant fish groups are as well.

Despite the number of factors included in model, the xyf-SOM was accurate in identifying and classifying fishing trips. The prediction percentage of the xyf-SOM ranged from a minimum of 51% for VL during the cold season in 2007 to a maximum of 100% in seven cases (2007 cold, 2011 cold, 2012 cold and 2009 warm for VL+SBL and 2007 cold, 2008 warm and 2011 warm for BL; Table 2). The BL fleet fishing trips were classified with the highest accuracy, with most groups up to 91%. The lowest prediction accuracy was observed for the VL groups from 51 to 72% (Table 2). The confusion matrix indicated that classification mistakes of the xyf-SOM were frequent within the same type of gear and not among different gear types (Table 2).

**Table 2:** Confusion matrix in percentage, reporting the performance of xyf-SOM in classifying new observations. BL fleet in blue, VL+SSBL in green, and VL in red; cold season is in light tones and warm season is in dark tones.

	Bottom longline											
	Cold						Warm					
Year	2007	2008	2009	2010	2011	2012	2007	2008	2009	2010	2011	2012
2007												
2010						1						
2012						1						
2007	**100**					2						
2007		4				1	**96**					6
2008		**88**				1						
2008			4					**100**		5		
2009			**88**									
2009			4						**95**	5		
2010				**100**								
2010					10	1			2	**80**		
2011		4			**90**		4					12
2012						**91**						
2012		4	4						2			**82**
	38	23	24	23	9	51	17	31	21	22	26	12
Bootstrap average % of prediction	**100**	**93**	**92**	**92**	**97**	**91**	**96**	**100**	**95**	**81**	**100**	**82**
and 95% CI	**93–100**	**87–98**	**85–98**	**85–98**	**90–100**	**84–98**	**91–100**	**96–100**	**89–99**	**51–100**	**83–100**	**76–89**
	Vertical-Line plus Shark longline											
	Cold						Warm					
Year	2007	2008	2009	2010	2011	2012	2007	2008	2009	2010	2011	2012
2007	**100**											
2007		4		4			**87**					
2008		**85**	3									
2008		4	3	11				**83**				
2009			**86**				9					
2009		4		4					**100**			
2010				**82**								
2010							4	17		**84**		
2011			3		**100**					5		
2012			3									
2012		4	3			**100**						**83**
2008										5		17
	4	27	31	28	24	8	15	21	20	16		8
Bootstrap average % of prediction	**100**	**85**	**85**	**82**	**100**	**100**	**87**	**83**	**100**	**84**		**83**
95% CI	**61–100**	**59–86**	**76–96**	**70–92**	**75–100**	**68–100**	**79–95**	**72–95**	**91–100**	**61–100**	****	**65–94**
	Vertical-Line											
	Cold						Warm					
Year	2007	2008	2009	2010	2011	2012	2007	2008	2009	2010	2011	2012
2007		3	2									
2007		3										
2008			2		7							
2008									13			
2010		3							13			
2007	**50**	3	2	14					13			
2007		3	5		7	12	**67**			19		
2008		**62**	2		13		13					8
2008		3			0	4		**73**		6		15
2009		3	**74**		7		7	18				8
2009	13	3	5						**60**			
2010	13	5	2	**71**			7					
2010	13		2			4	7	9		**56**		
2011		3		14	**67**							
2012		3				8						
2012	13	5	2			**72**				13		**69**
2010										6		
	6	32	34	11	23	18	12	20	26	29		4
95% CI	**34–68**	**48–75**	**62–85**	**58–86**	**56–77**	**59–88**	**51–80**	**61–84**	**48–73**	**37–74**	****	**51–82**

The xyf-SOMs for each gear separately did not show an improvement of the prediction rates. On the contrary, in the three cases prediction values were lower than those obtained in the general xyf-SOM (Table 3). However, the gear-specific xyf-SOM patterns were consistently similar to those found in the general map.

**Table 3:** Prediction percentages (mean ± 95% confidence interval) for the gear-specific supervised self-organized maps, based on 1000 bootstrap replicates. BL fleet in blue, VL+SSBL in green, and VL in red; cold season is in light tones and warm season is in dark tones.

Bottom longline											
Cold						Warm					
2007	2008	2009	2010	2011	2012	2007	2008	2009	2010	2011	2012
89.9	82.5	91.2	93.0	89.1	79.6	86.1	91.6	91.3	94.9	100.0	80.7
82.3–96.7	76.0–88.8	84.9–97.7	86.7–98.1	83.1–95.4	67.7–91.9	80.4–92.7	86.2–97.8	85.8–96.5	90.3–100	100–100	75.0–85.9
Vertical-Line plus Shark longline											
Cold						Warm					
2007	2008	2009	2010	2011	2012	2007	2008	2009	2010	2011	2012
91.9	87.7	86.3	76.0	91.5	90.7	86.0	82.0	91.3	84.3		94.3
81.0–100	79.8–93.3	86.7–96.2	79.8–92.7	85.8–97.7	67.7–100	81.0–96.2	67.7–83.9	84.3–97.3	72.4–91.0		89.9–100
Vertical-Line											
Cold						Warm					
2007	2008	2009	2010	2011	2012	2007	2008	2009	2010	2011	2012
61.0	68.4	61.5	66.0	63.5	41.0	62.5	47.9	54.8	70.4		57.0
41.0–84.3	60.5–76.6	53.6–69.3	53.6–77.5	54.6–73.2	17.7–67.7	51.0–73.7	37.4–56.6	43.9–66.0	62.9–78.3		46.0–68.9

### Species affinities

**Fig 6:**
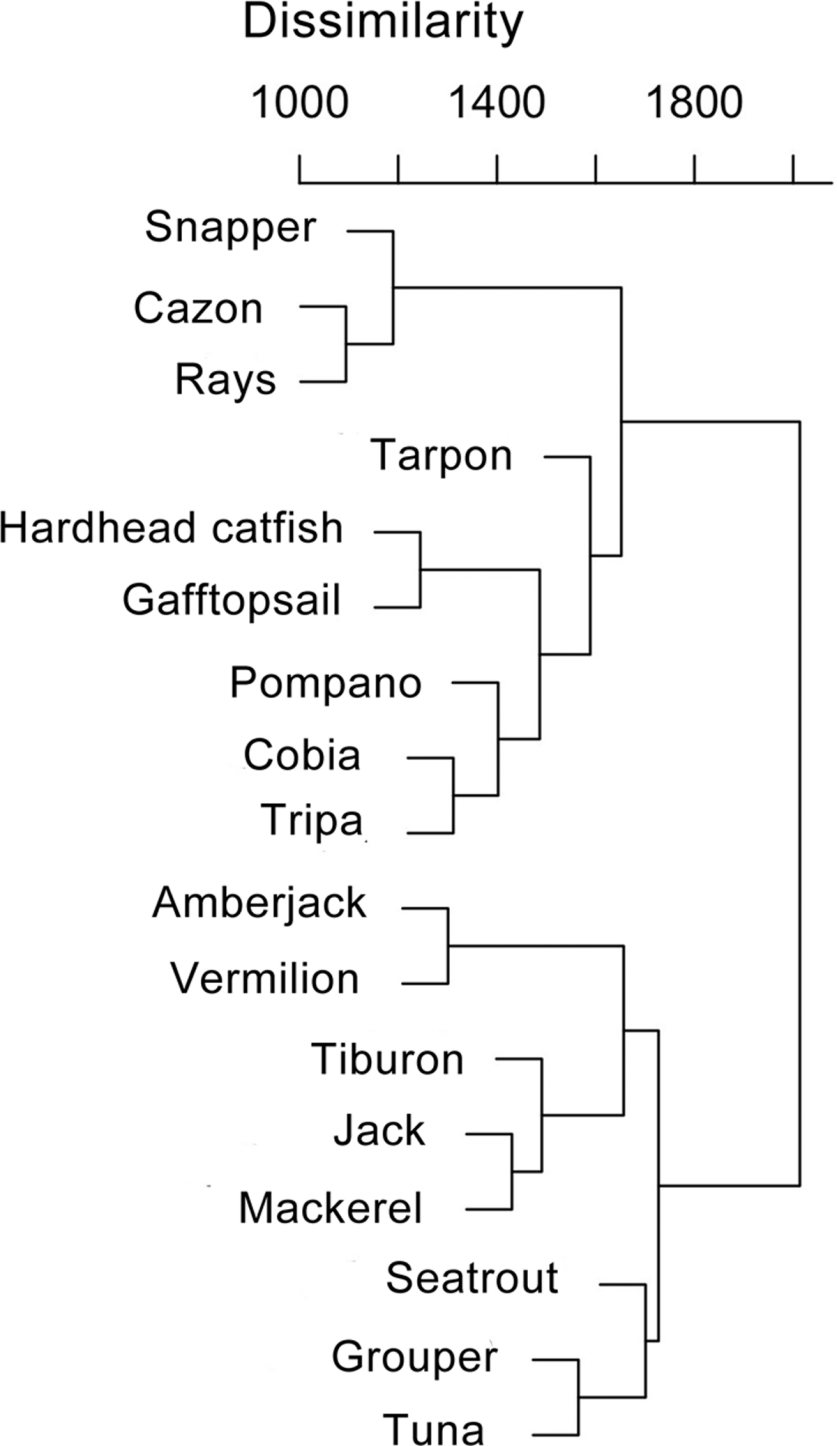
Hierarchical cluster analysis of fish groups’. Cluster was based on weights from the general xyf-SOM using Ward’s method.

The cluster analysis evidenced relationships between gear types and species resembling those revealed by the xyf-SOM (Fig 6). The cluster is divided in two principal branches. The upper branch is subdivided in two branches: one with three species associated with the VL fleet (snapper, cazon and rays) and one with species associated with the BL fleet (e.g. gafftopsail catfish, hardhead catfish). The lower principal branch contains species associated with VL+SBL fleet (e.g. tiburon, mackerel, Fig 6)

### Catch rate trends

**Fig 7:**
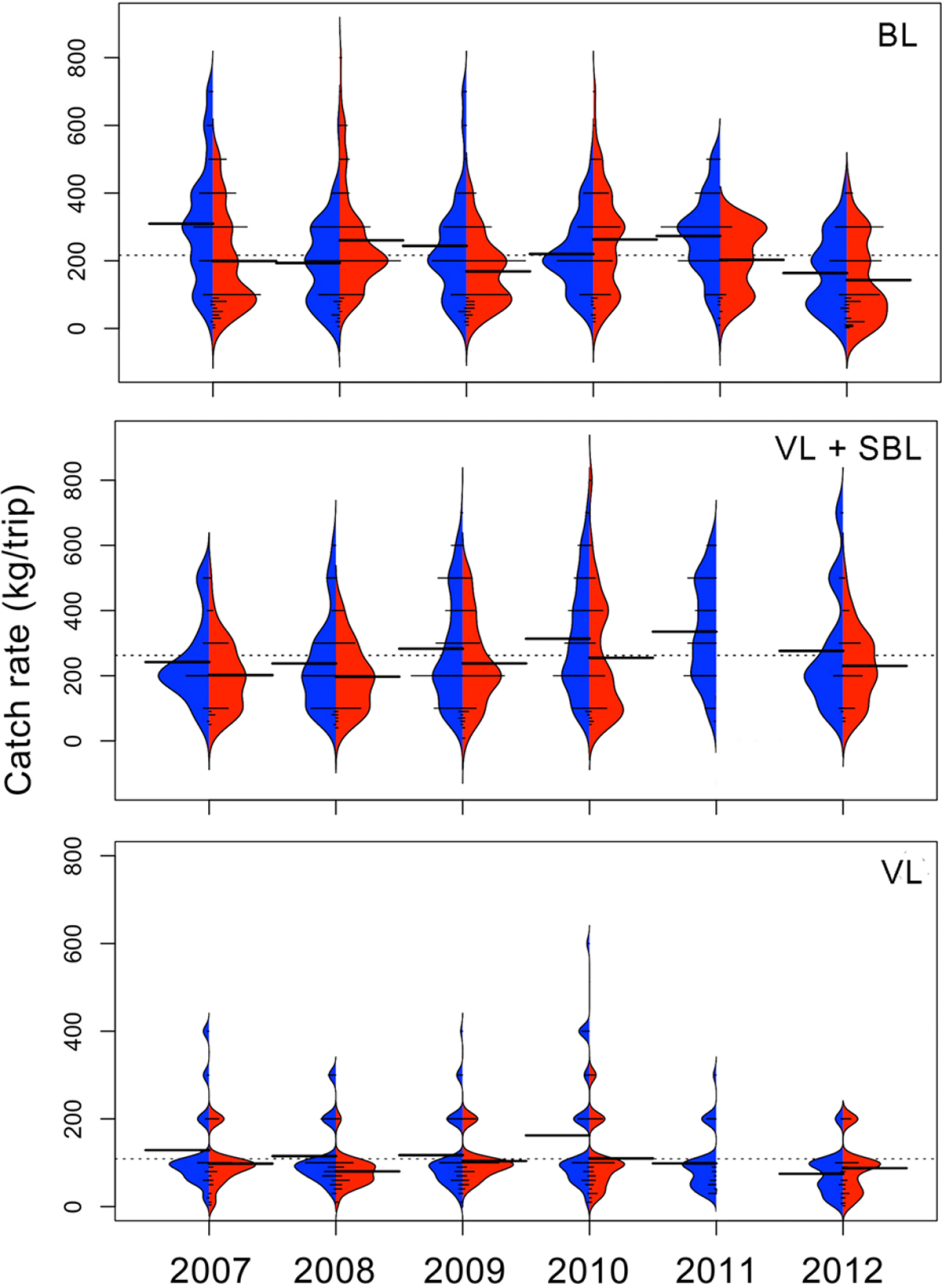
Seasonal catch rates (kg/trip) by fishing gear based on the weights derived from the xyf-SOM analysis. Cold seasons are in blue and warm seasons are in red. BL) bottom longline, VL+SBL) vertical line + shark bottom longline and, VL) vertical line. Dotted line indicates the overall mean catch rate; long thick lines indicate the mean catch rates by season; short lines indicate individual catch rates by node as derived from the xyf-SOM. Areas correspond to the kernel density shape.

Based on xyf-SOM weights, the BL fleet reached a mean total catch rate of 216.2 ± 139.2 (SD) kg/trip (Fig 7). The mean highest catch rates were found during the cold season of 2007 (309.5 ± 176.7 kg/trip) and the minimum value (143.16 ±115.08) was observed during the warm season of 2012 (Fig 7). Kernel density shapes indicate that individual catch rates were not distributed symmetrically around the seasonal mean catch rates (Fig 7). There were significant differences among years and seasons (Kruskal-Wallis H = 91.01, p < 0.001). Bonferroni *post hoc* tests indicated significant differences between 2007 cold season versus warm seasons of 2007, 2009, 2012 and the cold season of 2012 (p<0.001). Significant differences were also found between the warm season of 2012 versus warm seasons of 2008, 2010, and cold seasons of 2009, 2011 (p<0.001).

The Catch rate (kg/trip) behavior for VL + SBL fleet was similar than those of BL fleet, with an mean total catch rate of 262.4 ± 151.1 kg/trip (Fig 7). However, this may reflect the fact that the duration of trips with VL and SBL was higher (three days) than trips using BL (one day). Mean catch rates for the VL+SBL fleet was higher during cold season (Fig 7). Kernel density shapes indicated that individual catch rates were not distributed symmetrically around the seasonal mean (Fig 7). Significant differences between years were found (Kruskal-Wallis H = 43.52, p = 0.001). Bonferroni *post hoc* test indicated significant differences between the cold season of 2011 and the warm season of 2007, the cold and warm season of 2008 and the warm season of 2009 (p <0.01).

The lowest mean total catch rate was observed in the VL fleet (108.9 ± 78.0 kg/trip). The seasonal pattern of catch rate remained similar to the BL and VL+SBL fleets, so that catch rates were higher during cold season in most years, with the exception of 2012 (Fig 7). Kernel density shapes indicated that individual catch rates were not distributed symmetrically around the seasonal mean catch rates; rather, the kernel distribution was multimodal (Fig 7). No significant differences between years were found (Kruskal-Wallis H = 16.95 p = 0.07).

### Species-specific catch rate trends by gear

#### Bottom longline (BL)

**Fig 8:**
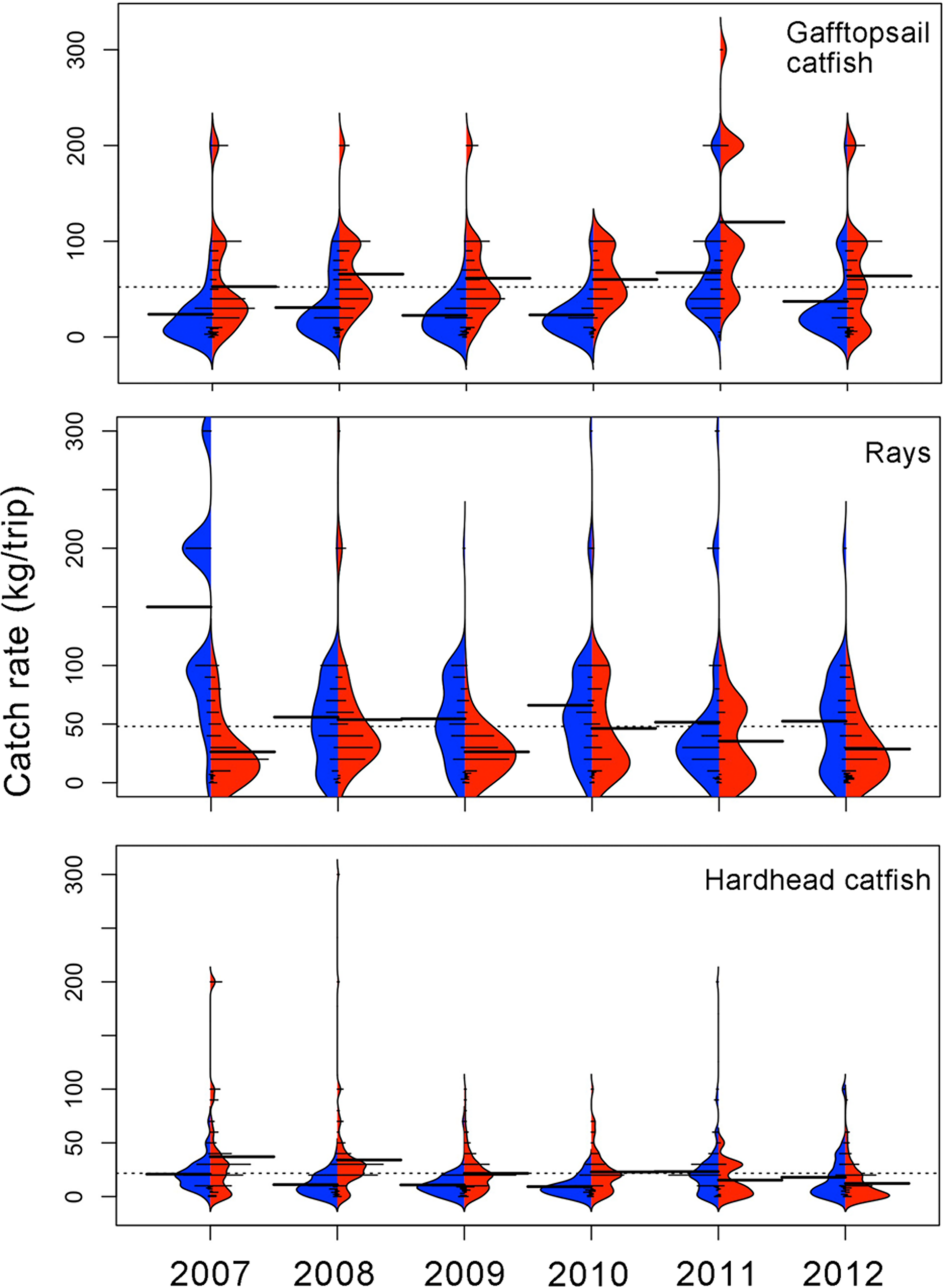
Seasonal catch rates (kg/ trip) for principal species associated with bottom longline (BL) based on the weights derived from the xyf-SOM analysis. Cold seasons are in blue and warm seasons are in red. Dotted line indicates the overall mean catch rate; long, thick lines indicate mean values by season; short lines indicate individual catch rates by node, as derived from the xyf-SOM. Areas correspond to the kernel density shape.

The overall mean catch rate for gafftopsail catfish was 52.2 ± 48.4 (SD) kg/trip (Fig 8). Catch rates were higher during the warm season in all years and also higher than the overall mean. The maximum value was observed during the warm season of 2011 (120.0 ± 80.1 kg·/trip/day). Despite the high mean values during warm seasons, kernel density shapes indicate that there was a high dispersion of individual catch rates and that most were lower than the mean value (Fig 8). Catch rates of gafftopsail catfish differed significantly between years and seasons (Kruskal-Wallis H = 167.41, p < 0.001). Bonferroni *post hoc* tests indicated significant differences (p < 0.001) between the warm season of 2011 with all seasons and years (p<0.001).

The second most important group in the BL fleet was the rays, reaching an overall mean of 48.0 ± 54.1 kg/trip. Contrary to the gafftopsail catfish, catch rates of rays were higher during cold seasons (Fig 8). Also here the kernel density shapes indicated that most catches were below this overall mean (Fig 8). It appears that there was a slight but consistent negative trend in rays catch rates during cold seasons, from its maximum catch rate (149.9 ± 100.9 kg/trip) during the cold season of 2007 to its minimum mean catch rate during the warm seasons of 2011 (35.3 ± 32.4 kg/trip) and 2012 (28.69 ± 25.7 kg/trip). Catch rates differed significantly between years and seasons (Kruskal-Wallis H = 141.96, p < 0.001). Bonferroni *post hoc* test indicated significant differences between the cold season of 2007 and all other seasons and years (p < 0.001).

Hardhead sea catfish had an overall mean catch rate of 21.7 ± 29.0 kg/trip (Fig 8). With the exception of 2011 and 2012 the higher mean catch rates of this species were found during warm seasons, and from 2007 to 2010. Significant differences between years and seasons were observed (Kruskal-Wallis H = 109.27, p < 0.001). Bonferroni *post hoc* test indicated significant seasonal differences (p < 0.001) between the warm season of 2007 and the cold season of 2008, between the warm season of 2008 and the cold season of 2009. Differences were also observed between the warm season of 2012 and the warm season of 2007 and 2008.

#### Vertical line plus shark bottom longline (VL+SBL)

**Fig 9:**
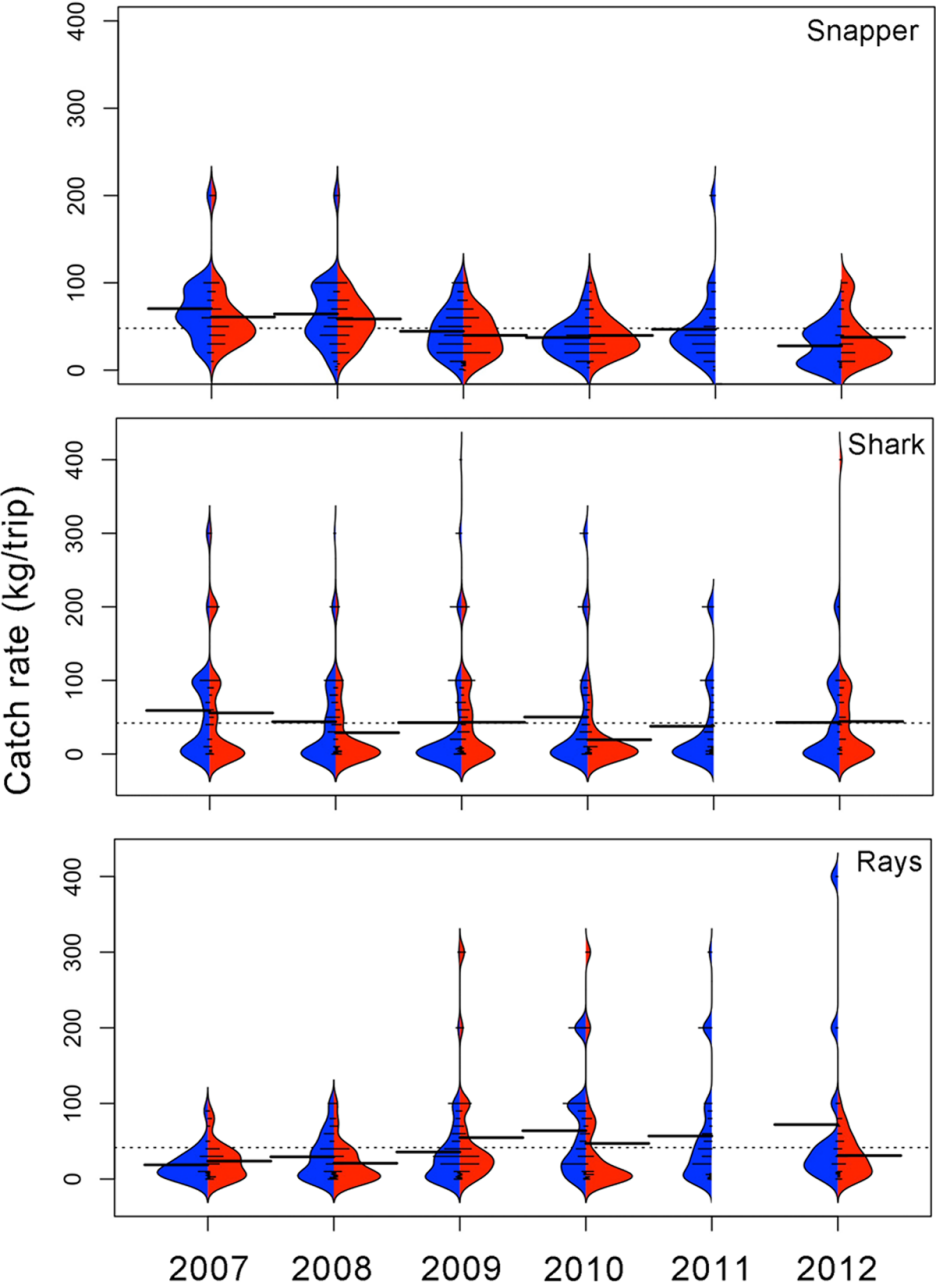
Seasonal catch rates (kg/ trip) for the main species associated to vertical line plus shark bottom longline (VL+SBL) based on weights derived from the seasonal SOM analysis. Cold seasons are in blue and warm seasons are in red. Dotted line indicates the overall mean catch rate; long, thick lines indicate the mean values by season, short lines indicate individual catch rates by node, as derived from the xyf-SOM. Areas correspond to kernel density shapes.

The overall mean catch rate for snappers was 47.9 ± 32.9 kg/trip (Fig 9). The highest mean catch rate was recorded during the cold (59.13 ± 74.2 kg/trip) and the warm (55.7 ± 76.3 kg/trip) seasons of 2007. In general catch rates of snapper were higher during cold than during warm season (Fig 9). There were significant differences between years and seasons (Kruskal-Wallis H = 64.77 p < 0.001). Bonferroni *post hoc* tests indicated significant differences (p < 0.001) between the cold season of 2010 and the cold and warm seasons of 2007, the cold and warm seasons of 2008.

In case of tiburon (sharks >20 kg) the overall mean catch rate values were lower than those of snappers (42.1 ± 68.3 kg/trip). Also, here there was a high dispersion of catch rate values, extreme values ranging from 200 to 400 kg/trip in all seasons and years, but with most fishing trips having below-overall mean, or even zero values (Fig 9). As with snapper, in this group higher catch rates were observed during the cold seasons. However, no significant differences between years and seasons were found for tiburon catch rates (Kruskal-Wallis H = 5.69 p = 0.84).

The third most abundant group associated with VL+SBL gears was the rays (overall mean = 41.8 ± 57.2 kg/trip). During the cold season an increase of the catch rates over time was observed, from a minimum of 18.8 ± 20.8 kg/trip during 2007 to a maximum of 72.0 ± 112.0 kg/trip during 2012 (Fig 9). Significant differences between years and seasons were found for ray catch rates (Kruskal-Wallis H = 34.49 p < 0.001). Bonferroni *post hoc* tests indicated significant differences (p < 0.001) between the cold season of 2008 and the cold season of 2010 only.

#### Vertical line (VL)

**Fig 10:**
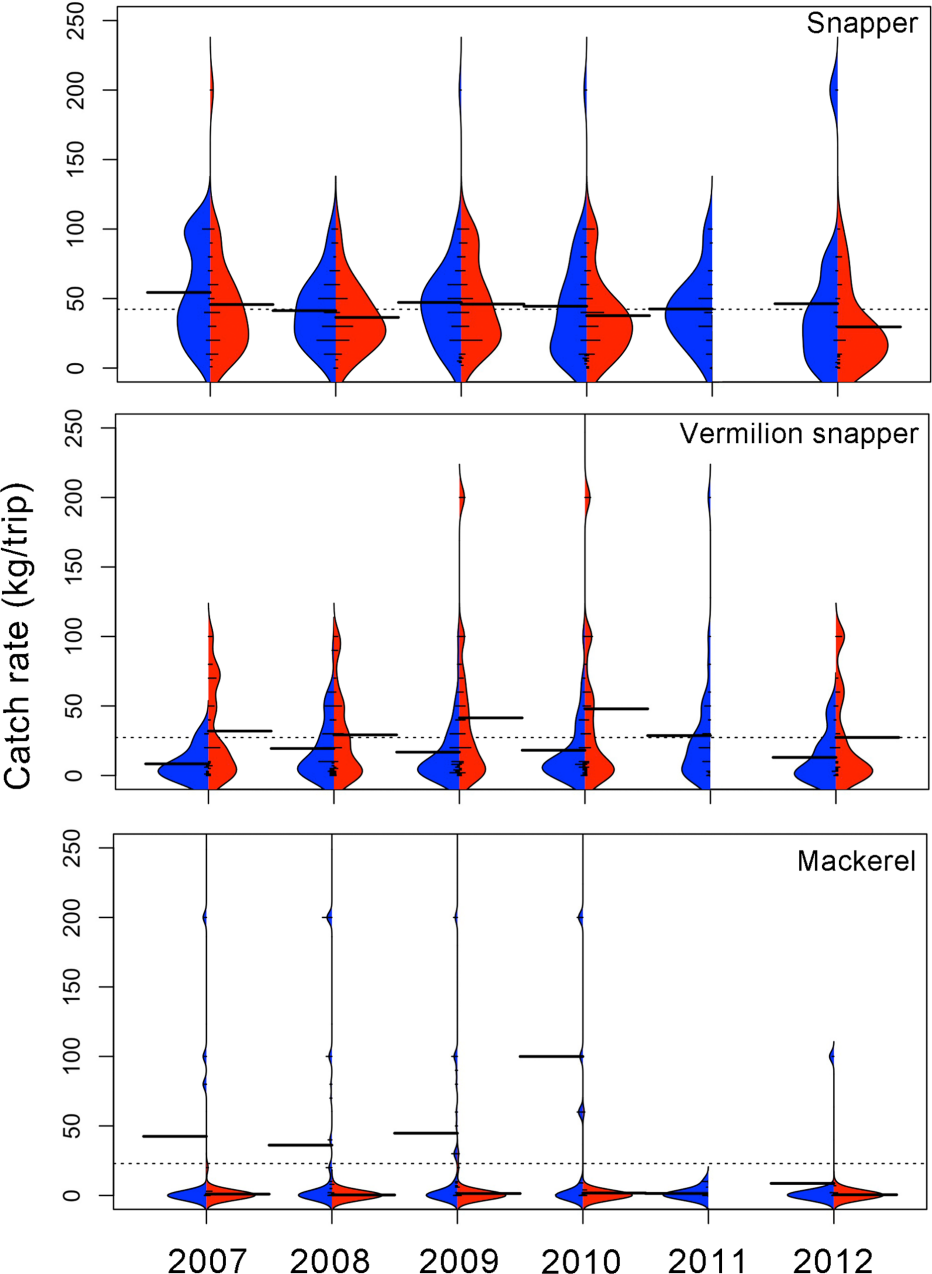
Seasonal catch rates (kg/trip) for the main species associated to vertical line (VL) based on weights derived from the seasonal SOM analysis. Cold seasons are in blue and warm seasons are in red. Dotted line indicates the overall mean catch rate; long, thick lines indicate the mean values by season, short lines indicate individual catch rates by node, as derived from the SOM. Areas correspond to kernel density shapes.

Since snapper is the principal target of the VL fleet, this species obtained the highest mean catch rate (overall mean = 42.3 ± 31.6 kg/trip). During almost all years and seasons the mean catch rate was near the overall mean, although the Kernel density function indicates that most of the fishing trips catch rates were below the overall mean (Fig 10). There were no significant differences between years and seasons (Kruskal-Wallis H = 19.13 p = 0.20).

In the case of vermilion snapper, the second most abundant species in the VL fleet, the overall mean catch rate (27.3 ± 38.9 kg/trip) was approximately a third less than the catch rate of snapper. Contrary to snapper, higher catch rates of vermilion were observed during warm seasons, especially during 2010 (48.0 ± 67.3 kg/trip) and 2009 (41.4 ± 52.8 kg/trip, Fig 10). There were significant differences between years and seasons (Kruskal-Wallis H = 19.13 p < 0.03). Bonferroni *post hoc* tests indicated significant differences (p < 0.01) between the warm season of 2010 and the warm season of 2009 and the cold season of 2010.

Mackerel, the third species of importance, showed variable catch rates with some extreme values (200 kg/trip approximately) that increased the mean catch rate (Fig 10). Nevertheless, the kernel density function indicates that most common catch rates were below the overall mean (23.1 ± 73.8 kg/trip, Fig 10). There were significant differences between years and seasons (Kruskal-Wallis H = 49.04 p < 0.001). Bonferroni *post hoc* tests indicated significant differences (p < 0.001) between the warm season of 2010 and the warm season of 2007, the cold and warm seasons of 2008, and the cold and warm seasons of 2009. Additionally, significant differences between the cold season of 2011 and the warm season of 2010 were observed.

## Discussion

The interpretation of the status and development of tropical small-scale fishery systems with traditional methods is usually difficult because of its high complexity caused by their multi-species and multi-gear nature [21, 52]. Traditional inference analyses in fisheries mostly concentrate on general trends based on mean catch rate values [68, 69]. However, these models rarely include the sources of variability in their estimates. Variability in catch rates is caused by climatic and oceanographic variation, species diversity and seasonal inter-annual variability, but it also depends on the behavior of individual fishermen or on the individual fishing trip, which produces a high degree of uncertainty in the prediction models [70, 71].

Therefore, the principal focus of this study was to infer the effect of the individual fishing trips on the general trends in catch rates, taking into account the target species and environmental variation. Since conventional analyses are limited in dealing with such variability [1, 72], we used SOM, an artificial neural network, which allowed us to visualize the individual contribution of fishing trips to general trends. Furthermore, SOM analysis allowed us to recognize the internal behavior of the fleet in relation to target species, and the effect of seasons and inter-annual variation on catch rates [28].

Despite the strong similarity of vessels and gears in the analyzed fishery, the internal variability of the fleets did not reflect this homogeneity. Heterogeneity between fishing trips has been attributed to factors such as personal goals, experience, economic constraints, and environmental limitations [3, 70, 73]. Some of these factors, related to decision-making and learning process of fishermen have been successfully simulated with neural networks. Results of such simulations indicate that fishermen will try to avoid risky or uncertain decisions, but also that reliable decisions do not always result in high catch rates [73]. This could be interpreted as the natural uncertainty of the fishery process, which is reflected in our SOM models.

In our case, and based on the xyf-SOM results, we can infer that fishermen using BL are more dependent on changes in species composition with seasons and along years, since fishermen do not have target species. In contrast, in the VL and VL+SBL fleets, both of which have target species (snappers and sharks respectively), the variation among years and seasons is more dependent on the fishermen’s experience and knowledge of the fishery area [3, 70].

Neural networks, with their high flexibility and non-linear nature, are an effective form of incorporating and interpreting the natural variability of small-scale fisheries [19, 21]. In this way, the use of xyf-SOM allowed us to obtain an accurate picture of the variability within the San Pedro port small-scale fishery fleet, reflecting the heterogeneity between fishing trips, gears, seasons and years. It is important to note that not all the fishing trips are targeting the most abundant species, and therefore the species composition is not homogeneous between fishing trips, nor are the individual catch rates normally distributed. A similar behavior has been observed in the flatfish fishery of the North Sea, where some fishermen are more dependent on flatfish than others that have the flexibility to fish on other species [74]. Differences in performance among fishermen may depend on their flexibility to switch to non-target species in case of low abundance or absence of the main target species [18, 74]. In our case, hardhead catfish and rays for BL and vermilion snapper for VL+SBL represent such important supplementary fish groups.

Besides the useful graphical representation provided by the SOM, the use of SOM weights for posterior analyses has been shown to be advantageous for pattern interpretations with significant benefits in complex problems [23, 32, 75]. In our study, xyf-SOM weights were applied to analyze species’ affinities with particular gears by cluster analysis, but also to visualize the variability of the catch rate within the fleets by means of beanplots [63, 76]. Based on xyf-SOM analyses applied on the landing composition at functional group level, temporal and spatial patterns of industrial fisheries were identified within large marine ecosystems, revealing a shift in the composition of landings, and enhancing the mixed composition of these landings [23].

The predictive power is critical in fishery models [77]. Since the xyf-SOM technique presents a high capacity of classification of multidimensional data [32], our prediction values were high with narrow confidence intervals, although prediction percentages were lower in the VL xyf-SOMs than in the BL and VL+SBL xyf-SOMs. The lower predictive power of the former could be attributed to the high similarity in the species composition of fishing trips in VL, caused by its principal target fish group, snappers, being present in 100% of all fishing trips. Despite this greater similarity in species composition this does not necessarily result in a reduction in the variability of catch rates, nor in the total catch rate, again showing the strong effect of the behavior of individual fishermen [69].

The seasonal differences in catch rates were another interesting aspect detected by the SOM. Seasonal changes in catch rates could reflect abundance changes in relation with life-cycle phases [78, 79]. For instance, gafftopsail catfish showed a clear seasonal variation in its abundances. During the warm season (May-October) this species comes near the coast for reproduction [35, 80]. This is reflected in an increase of its catch rates during warm season. By contrast, during the cold months (November-April), gafftopsail catfish is offshore, and consequently, the BL fleet operating near the shore, has lower catch rates. These results highlight that interactions between fisheries and the life cycle and reproductive strategy of this species - such as a marked reproductive season, low fecundity and estuarine dependence - need to be taken into account for the sustainable exploitation of this important species [81].

Another group with a markedly seasonal behavior corresponds to the elasmobranch species [34]. The Mexican Gulf of Mexico elasmobranch fishery is composed of at least 18 species [47, 82]. Although sharks were not analyzed per species, but in three size groups, the xyf-SOM detected a clear association in the abundance patterns of tiburon (sharks >20 kg) and the medium-sized (2–20 kg) cazon group. Especially for the VL+SBL fishing gears, higher catch rates of both groups were located in adjacent areas inside the SOM, indicating this association. This possibly reflects seasonal migratory movements of sharks [83]. Additionally, sharks were only present in high abundances during certain years: cazon during 2008, 2009 and 2011 and tiburon during 2007, 2009 and 2012.

Although elasmobranchs do not present a high proportion of the landings of the San Pedro port fleet, there appears to be a seasonal increase in the occurrence and catch rates of neonate and juvenile sharks (tripa and cazon) in the BL and VL fleet. The effect of such catches for the populations has not been evaluated. However, models for populations of *Rhizoprionodon taylori*, *Squalus acanthias* and *Dipturus batis* based on Leslie matrix, conclude that mortality of neonates and juveniles of the year has relatively little effect on population growth rates [84, 85]. These models indicate a stronger risk of depletion with the fraction of reproductive potential being removed annually, i.e. with fishing pressure on the oldest ages having the highest reproductive potential.

The southern stingray *Dasyatis americana* is one of the most abundant species of the marine small-scale fisheries of Tabasco and Campeche states [43, 86]. Our results corroborate this: southern stingray is second in importance of the total catch volume, and the most important elasmobranch. The high importance of this species is potentially related to two factors: a) the decrease in shark abundance in the study area may have caused the increase of other species with lower trophic levels [87, 88] and b) since the decrease of sharks, stingray became economically more important [43]. Changes in fishery patterns are reflected in the official statistics, which show a strong decrease of sharks since 1995 and an increase in rays since 2002 [33]. This interpretation should be taken with caution, because before 1997 rays were not included in the official statistics, possibly being included in the shark category [43]. In addition, the high occurrence of gravid females of the southern stingray in the small-scale fishery of San Pedro port indicates a potential reproduction area [86] and the slight decreasing trend in catch rates suggests the necessity of more research on management issues for the sustainability of this fishery.

The low effort applied by trip by the BL fleet (one-day trips vs. two to three-day trips), while still obtaining higher catch rates, compared to the VL fleet apparently favored the former. However, the economic value of the fish groups caught by VL is usually higher. While the prices of gafftopsail catfish, which is mostly caught by BL varied from 3 to 15 Mexican pesos (0.21–1.09 USD) by eviscerated kilogram during 2007–2010 years, the price of the snappers varied from 17 to 70 Mexican pesos (1.23–5.09 USD) by eviscerated kilogram in the same period (Mendoza-Carranza unpublished data). This apparent difference in profit has an effect on the economic and safety risks that fishermen take [89, 90]. Piniella and Fernández-Engo [91] mention this relationship for artisanal fishermen of Andalucia, Spain, where economic gains lead to prolonged working days and an increment of the injury risk factors. While the BL fleet has a restricted fishing time (12–14 hours), and a fishing area close to the coast, the VL fleet has a longer fishing time (two to three days) and an extensive fishing area further offshore, implying more safety risks and monetary investment. These differences are especially clear since both fleets use the same vessel type (7m fiberglass vessel with 60–110 hp outboard motor). Furthermore, the socio-economy of small-scale fisheries is inadequately understood [92, 93] and there is an urgent need to determine the best long- and short-term management strategies based on accurate mathematical and economical models [94].

The xyf-SOM analysis shows that species composition varied greatly within these multispecies fisheries and that this important for understanding the variability of catch rates. Differences in catch composition between fishermen might have a relation with the spatial allocation of resources and individual fishermen [94] and the complex oceanographic conditions. Despite the apparent homogeneity of the fishing area, it presents some characteristics that could affect the preferences of fishermen for a particular area. A high productivity characterizing this fishing area is especially related with the discharge of the Grijalva-Usumacinta river basin (120,000 million m3/year) [42]. The high discharge of water from Grijalva-Usumacinta basin provides a significant input of detritus that sustains a food web with a high degree of omnivory with intricate relationships in a well-organized and resilient ecosystem [95].

Additionally discharge fluctuations among years are related to seasonal changes in the salinity of the coastal water [37, 42]. A coastal upwelling process, and seasonal changes in the coastal currents are present in our study area [37, 40, 96]. All these aspects result in a dynamic oceanographic pattern, which is not yet well known, but which influences the species composition of the catches and the fisheries trends. Therefore, it is necessary to perform more detailed studies of the spatial allocation of effort by fishermen in our study area to further understand the fleet dynamics [69].

## References

[1] Hollowed AB, Bax N, Beamish R, Collie J, Fogarty M, Livingston P, et al Are multispecies models an improvement on single-species models for measuring fishing impacts on marine ecosystems? ICES J Mar Sci. 2000;57(3):707–19.

[2] Dreyfus-Leon M, Kleiber P. A spatial individual behaviour-based model approach of the yellowfin tuna fishery in the eastern Pacific Ocean. Ecol Model. 2001;146(1):47–56.

[3] Wilen JE, Smith MD, Lockwood D, Botsford LW. Avoiding Surprises: Incorporating Fisherman Behavior into Management Models. Bull Mar Sci. 2002;70:553–75.

[4] Brosse S, Giraudel JL, Lek S. Utilisation of non-supervised neural networks and principal component analysis to study fish assemblages. Ecol Model. 2001;146(1):159–66.

[5] Winker H, Kerwath SE, Attwood CG. Comparison of two approaches to standardize catch-per-unit-effort for targeting behaviour in a multispecies hand-line fishery. Fish Res. 2013;139:118–31.

[6] Keyl F, Wolff M. Environmental variability and fisheries: what can models do? Rev Fish Biol Fish. 2008;18(3):273–99. doi: 10.1007/s11160-007-9075-5

[7] Hsieh C-h Glaser SM, Lucas AJ, Sugihara G. Distinguishing random environmental fluctuations from ecological catastrophes for the North Pacific Ocean. Nature. 2005;435(7040):336–40. http://www.nature.com/nature/journal/v435/n7040/suppinfo/nature03553_S1.html. doi: 10.1038/nature03553 15902256 10.1038/nature03553

[8] Galindo-Cortes G, De Anda-Montañez JA, Arreguín-Sánchez F, Salas S, Balart EF. How do environmental factors affect the stock–recruitment relationship? The case of the Pacific sardine (Sardinops sagax) of the northeastern Pacific Ocean. Fish Res. 2010;102(1):173–83.

[9] Bertrand S, Bertrand A, Guevara-Carrasco R, Gerlotto F. Scale-invariant movements of fishermen: the same foraging strategy as natural predators. Ecol Appl. 2007;17(2):331–7. 17489242 10.1890/06-0303

[10] Vermard YVY, Marchal PMP, Mahévas SMS, Thébaud OTO. A dynamic model of the Bay of Biscay pelagic fleet simulating fishing trip choice: the response to the closure of the European anchovy (*Engraulis encrasicolus*) fishery in 2005. Can J Fish Aquat Sci. 2008;65(11):2444–53.

[11] Béné C. Small-scale fisheries: assessing their contribution to rural livelihoods in developing countries. 2006.

[12] Salas S, Chuenpagdee R, Seijo JC, Charles A. Challenges in the assessment and management of small-scale fisheries in Latin America and the Caribbean. Fish Res. 2007;87(1):5–16. https://doi.org/10.1016/j.fishres.2007.06.015.

[13] Cotter AJR, Pilling GM. Landings, logbooks and observer surveys: improving the protocols for sampling commercial fisheries. Fish Fish. 2007;8(2):123–52. doi: 10.1111/j.1467-2679.2007.00241.x

[14] Russo T, Carpentieri P, Fiorentino F, Arneri E, Scardi M, Cioffi A, et al Modeling landings profiles of fishing vessels: An application of Self-Organizing Maps to VMS and logbook data. Fish Res. 2016;181:34–47.

[15] Walsh WA, Bigelow Ka, Sender KL. Decreases in Shark Catches and Mortality in the Hawaii-Based Longline Fishery as Documented by Fishery Observers. Marine and Coastal Fisheries. 2009;1(1):270–82. doi: 10.1577/C09-003.1

[16] Walsh WA, Ito RY, Kawamoto KE, McCracken M. Analysis of logbook accuracy for blue marlin (Makaira nigricans) in the Hawaii-based longline fishery with a generalized additive model and commercial sales data. Fish Res. 2005;75(1–3):175–92. https://doi.org/10.1016/j.fishres.2004.11.007.

[17] Deporte N, Ulrich C, Mahévas S, Demanèche S, Bastardie F. Regional métier definition: a comparative investigation of statistical methods using a workflow applied to international otter trawl fisheries in the North Sea. ICES Journal of Marine Science: Journal du Conseil. 2012:fsr197.

[18] Pradhan NC, Leung P. Modeling trip choice behavior of the longline fishers in Hawaii. Fish Res. 2004;68(1):209–24.

[19] Hillary RM. Practical uses of non-parametric methods in fisheries assessment modelling. Mar Freshwater Res. 2012;63(7):606–15.

[20] Giraudel JL, Lek S. A comparison of self-organizing map algorithm and some conventional statistical methods for ecological community ordination. Ecol Model. 2001;146(1–3):329–39. https://doi.org/10.1016/S0304-3800(01)00324-6.

[21] Suryanarayana I, Braibanti A, Sambasiva Rao R, Ramam VA, Sudarsan D, Nageswara Rao G. Neural networks in fisheries research. Fish Res. 2008;92(2):115–39.

[22] Chon T-S. Self-Organizing Maps applied to ecological sciences. Ecol Inform. 2011;6(1):50–61. https://doi.org/10.1016/j.ecoinf.2010.11.002.

[23] Conti L, Grenouillet G, Lek S, Scardi M. Long-term changes and recurrent patterns in fisheries landings from Large Marine Ecosystems (1950–2004). Fish Res. 2012;119:1–12.

[24] Gaertner D, Dreyfus-Leon M. Analysis of non-linear relationships between catch per unit effort and abundance in a tuna purse-seine fishery simulated with artificial neural networks. ICES J Mar Sci. 2004;61(5):812–20.

[25] Glaser SM, Ye H, Maunder M, MacCall A, Fogarty M, Sugihara G. Detecting and forecasting complex nonlinear dynamics in spatially structured catch-per-unit-effort time series for North Pacific albacore (*Thunnus alalunga*). Can J Fish Aquat Sci. 2011;68(3):400.

[26] Kohonen T. Essentials of the self-organizing map. Neural Networks. 2013;37:52–65. doi: 10.1016/j.neunet.2012.09.018 23067803 10.1016/j.neunet.2012.09.018

[27] Kohonen T. Self-organizing maps: Springer; 2001.

[28] Hyun K, Song MY, Kim S, Chon TS. Using an artificial neural network to patternize long-term fisheries data from South Korea. Aquat Sci. 2005;67(3):382–9.

[29] Albañez-Lucero MO, Arreguín-Sánchez F. Modelling the spatial distribution of red grouper (Epinephelus morio) at Campeche Bank, México, with respect substrate. Ecol Model. 2009;220(20):2744–50. https://doi.org/10.1016/j.ecolmodel.2009.07.007.

[30] Simić VM, Simić SB, Stojković Piperac M, Petrović A, Milošević D. Commercial fish species of inland waters: A model for sustainability assessment and management. Sci Total Environ. 2014;497–498:642–50. https://doi.org/10.1016/j.scitotenv.2014.07.092. 25170830 10.1016/j.scitotenv.2014.07.092

[31] Melssen W, Wehrens R, Buydens L. Supervised Kohonen networks for classification problems. Chemometrics Intellig Lab Syst. 2006;83(2):99–113.

[32] Papadimitriou S, Mavroudi S, Vladutu L, Pavlides G, Bezerianos A. The supervised network self-organizing map for classification of large data sets. Appl Intell. 2002;16(3):185–203.

[33] SAGARPA-CONAPESCA. Anuario estadístico de acuacultura y pesca 2011. Mazatlán: CONAPESCA, 2011.

[34] Fernández JI, Álvarez-Torres P, Arreguín-Sánchez F, López-Lemus LG, Ponce G, <Díaz-de-León A, et al. Coastal Fisheries in Mexico. In: S. Salas RC, A. Charles and J.C. Seijo., editor. Coastal fisheries of Latin America and the Caribbean. FAO Fisheries and Aquaculture Technica Paper. 544. Rome: FAO Fisheries and Aquaculture Technica Paper; 2011. p. 231–84.

[35] Mendoza-Carranza M, Romero-Rodríguez A, Arévalo-Frías W, Segura-Berttolini EC, Ramirez-Mosqueda E. El bagre bandera *Bagre marinus* (Mithcill, 1815) como especie clave de la pesca marina de pequeña escala en la costa de Tabasco In: Sánchez AJ, Chiappa-Carrara X, Pérez B, editors. Recursos Acuáticos Costeros del Sureste: Tendencias actuales en investigación y estado del arte. 2. México, D.F: RECORECOS, CONCYTEY, UNACAR, UJAT, ECOSUR, UNAM; 2012 p. 527–47.

[36] Cram S, de León CAP, Sommer I, Miceli S, Fernández P, Rivas H, et al Metal distribution in coral reef complex Cayo Arcas in the Gulf of Mexico. Environ Monit Assess. 2009;151(1–4):413–24. doi: 10.1007/s10661-008-0285-7 18563611 10.1007/s10661-008-0285-7

[37] Monreal-Gómez MA, Salas-de-León DA, Velasco-Mendoza H, Caso M, Pisanty I, Ezcurra E. La hidrodinámica del Golfo de México In: Caso M, Pisanti I, Ezcurra E, editors. Diagnóstico ambiental del Golfo de México. 1. México, D.F: Instituto Nacional de Ecología, SEMARNAT; 2004 p. 47–68.

[38] Arévalo-Frías W, Mendoza-Carranza M. Larvas y juveniles de peces en ambientes estuatinos de la Reserva de la Biosfera Pantanos de Centla y su zona adyacente In: Sánchez AJ, Chiappa-Carrara X, Pérez B, editors. Recursos Acuáticos Costeros del Sureste: Tendencias actuales en investigación y estado del arte. 2. México, D.F: RECORECOS, CONCYTEY, UNACAR, UJAT, ECOSUR, UNAM; 2012 p. 242–69.

[39] ERDDAP—The Environmental Research Division's Data Access Program. [Internet]. NOAA. 2011. Available from: http://coastwatch.pfeg.noaa.gov/erddap.

[40] Zavala-Hidalgo J, Gallegos-García A, Martínez-López B, Morey SL, O’Brien JJ. Seasonal upwelling on the western and southern shelves of the Gulf of Mexico. Ocean Dynam. 2006;56(3–4):333–8.

[41] Zavala‐Hidalgo J, Morey SL, O'Brien JJ. Seasonal circulation on the western shelf of the Gulf of Mexico using a high‐resolution numerical model. Journal of Geophysical Research: Oceans (1978–2012). 2003;108(C12). doi: 10.1029/2003JC001879

[42] Yáñez-Arancibia A, Day JW, Currie-Alder B. Functioning of the Grijalva-Usumacinta river delta, Mexico: Challenges for coastal management. Ocean Yearb. 2009;23:473–501.

[43] Pérez-Jiménez JC, Méndez-Loeza I, Mendoza-Carranza M, Cuevas-Zimbron E. Análisis histórico de las pesquerías de elasmobranquios del sureste del Golfo de México In: Sánchez AJ, Chiappa-Carrara X, Peréz B, editors. Recursos Acuáticos Costeros del Sureste: Tendencias actuales en investigación y estado del arte. 2. México, D.F: RECORECOS, CONCYTEY, UNACAR, UJAT, ECOSUR, UNAM; 2012 p. 463–81.

[44] Diario Oficial de la Federación. PROYECTO de Norma Oficial Mexicana PROY-NOM-060-PESC-2011, Pesca responsable en cuerpos de aguas continentales dulceacuícolas de jurisdicción federal de los Estados Unidos Mexicanos Especificaciones para el aprovechamiento de los recursos pesqueros. Mexico, D.F.: Gobierno Federal; 2013.

[45] Arreguín-Sánchez F, Contreras M, Moreno V, Burgos R, Valdés R, Arreguín-Sánchez F, et al., editors. Population dynamics and stock assessment of red grouper (Epinephelus morio) fishery on Campeche Bank, Mexico1996 1996.

[46] Arreguin-Sanchez F, Zetina-Rejón M, Manickchand-Heileman S, Ramırez-Rodrıguez M, Vidal L. Simulated response to harvesting strategies in an exploited ecosystem in the southwestern Gulf of Mexico. Ecol Model. 2004;172(2):421–32. https://doi.org/10.1016/j.ecolmodel.2003.09.016.

[47] Castillo-Géniz JL, Marquez-Farias JF, Cruz MC, Rodriguez de la Cruz E, Cid del Prado A. The Mexican artisanal shark fishery in the Gulf of Mexico: towards a regulated fishery. Mar Freshwater Res. 1998;49(7):611–20.

[48] Hoese HD, Moore RH. Fishes of the Gulf of Mexico, Texas, Louisiana, and adjacent waters. 1977.

[49] Reséndez MA. Catálogo de las especies acuáticas de importancia comercial en el Estado de Tabasco, artes y métodos de pesca. Villahermosa, Tabasco, México: Secretaria de Desarrollo, Gobierno del Estado de Tabasco; 1998.

[50] Carpenter KE. The living marine resources of the western central Atlantic. Carpenter KE, editor. Rome: FAO; 2002 773 p.

[51] Nelson JS, Crossman EJ, Espinosa-Pérez H, Findley LT, Gilbert CR, Lea RN, et al Common and scientific names of fishes from the United States, Canada and Mexico: American Fisheries Society; 2004.

[52] Tsehaye I, Machiels MAM, Nagelkerke LAJ. Rapid shifts in catch composition in the artisanal Red Sea reef fisheries of Eritrea. Fish Res. 2007;86(1):58–68.

[53] Hothorn T, Hornik K, Strobl C, Zeileis A. Party: A laboratory for recursive partytioning. Citeseer; 2010.

[54] Hothorn T, Hornik K, Zeileis A. Unbiased recursive partitioning: A conditional inference framework. J Comput Graph Stat. 2006;15(3):651–74.

[55] Kohonen T. The self-organizing map. Proceedings of the IEEE. 1990;78(9):1464–80.

[56] Kohonen T, Hynninen J, Kangas J, Laaksonen J. Som pak: The self-organizing map program package. Report A31, Helsinki University of Technology, Laboratory of Computer and Information Science. 1996.

[57] Wehrens R, Buydens LMC. Self-and super-organizing maps in R: the Kohonen package. J Stat Softw. 2007;21(5):1–19.

[58] Ejarque-Gonzalez E, Butturini A. Self-organising maps and correlation analysis as a tool to explore patterns in excitation-emission matrix data sets and to discriminate dissolved organic matter fluorescence components. PLoS ONE. 2014;9(6):e99618 doi: 10.1371/journal.pone.0099618 24906009 10.1371/journal.pone.0099618 PMC4048288

[59] Park Y-S, Céréghino R, Compin A, Lek S. Applications of artificial neural networks for patterning and predicting aquatic insect species richness in running waters. Ecol Model. 2003;160(3):265–80.

[60] Jutagate T, Lek S, Sawusdee A, Sukdiseth U, Thapanand‐Chaidee T, Thongkhoa S, et al Spatio‐temporal variations in fish assemblages in a tropical regulated lower river course: an environmental guild. River Res Appl. 2011;27(1):47–58.

[61] Tudesque L, Gevrey M, Grenouillet G, Lek S. Long-term changes in water physicochemistry in the Adour–Garonne hydrographic network during the last three decades. Water Res. 2008;42(3):732–42. doi: 10.1016/j.watres.2007.08.001 18023839 10.1016/j.watres.2007.08.001

[62] DiCiccio T, J, Bradley E. Bootstrap confidence interval. Stat Sci. 1996;11(3):189–228.

[63] Kampstra P. Beanplot: A boxplot alternative for visual comparison of distributions. Jour; 2008.

[64] Venables WN, Ripley BD. Modern applied statistics with S: Springer; 2002.

[65] Sokal RR, Rohlf FJ. Biometry: the principles and practice of statistics in biological research. New York: W. H. Freeman; 1995 887 p.

[66] Zar JH. Biostatistical analysis. New Jersey: Prentice-Hall; 2010 xiii, 944 p. p.

[67] Siegel S, Castellan NJ. Nonparametric statistics for the behavioral sciences. New York: McGraw-hill; 1988 399 p.

[68] Caddy JF, Seijo JC. This is more difficult than we thought! The responsibility of scientists, managers and stakeholders to mitigate the unsustainability of marine fisheries. Philos T Roy Soc B. 2005;360(1453):59–75. 10.1098/rstb.2004.1567 PMC1636105 15713588

[69] Maunder MN, Sibert JR, Fonteneau A, Hampton J, Kleiber P, Harley SJ. Interpreting catch per unit effort data to assess the status of individual stocks and communities. ICES J Mar Sci. 2006;63(8):1373–85.

[70] Branch TA, Hilborn R, Haynie AC, Fay G, Flynn L, Griffiths J, et al Fleet dynamics and fishermen behavior: lessons for fisheries managers. Can J Fish Aquat Sci. 2006;63(7):1647–68.

[71] Salas S, Gaertner D. The behavioural dynamics of fishers: management implications. Fish Fish. 2004;5(2):153–67. doi: 10.1111/j.1467-2979.2004.00146.x.

[72] Ulrich C, Le Gallic B, Dunn MR, Gascuel D. A multi-species multi-fleet bioeconomic simulation model for the English Channel artisanal fisheries. Fish Res. 2002;58(3):379–401. https://doi.org/10.1016/S0165-7836(01)00393-9.

[73] Dreyfus-León JM. Individual-based modelling of fishermen search behaviour with neural networks and reinforcement learning. Ecol Model. 1999;120(2):287–97.

[74] Andersen BS, Vermard Y, Ulrich C, Hutton T, Poos JJ. Challenges in integrating short-term behaviour in a mixed-fishery Management Strategies Evaluation frame: A case study of the North Sea flatfish fishery. Fish Res. 2010;102(1):26–40.

[75] Kruk A. Self-organizing maps in revealing variation in non-obligatory riverine fish in long-term data. Hydrobiologia. 2006;553(1):43–57.

[76] Drago C, Lauro C, Scepi G. Visualization and Analysis of Large Datasets by Beanplot PCA. 2013.

[77] Fulton EA, Smith ADM, Johnson CR. Effect of complexity on marine ecosystem models. Mar Ecol Prog Ser. 2003;253:1–16.

[78] Petitgas P, Secor DH, McQuinn I, Huse G, Lo N. Stock collapses and their recovery: mechanisms that establish and maintain life-cycle closure in space and time. ICES J Mar Sci. 2010;67(9):1841–8.

[79] Tian Y, Akamine T, Suda M. Modeling the influence of oceanic-climatic changes on the dynamics of Pacific saury in the northwestern Pacific using a life cycle model. Fish Oceanogr. 2004;13:125–37. doi: 10.1111/j.1365-2419.2004.00314.x.

[80] Mendoza-Carranza M, Hernández-Franyutti A. Annual reproductive cycle of gafftopsail catfish, *Bagre marinus* (Ariidae) in a tropical coastal environment in the Gulf of Mexico. Hidrobiologica. 2005;15(3):275–82.

[81] Segura-Berttolini EC, Mendoza-Carranza M. Importance of male gafftopsail catfish, *Bagre marinus* (Pisces: Ariidae), in the reproductive process. Cienc Mar. 2013;39(1):29–39.

[82] Bonfil R. Status of shark resources in the Southern Gulf of Mexico and Caribbean: implications for management. Fish Res. 1997;29(2):101–17.

[83] Speed CW, Field IC, Meekan MG, Bradshaw C. Complexities of coastal shark movements and their implications for management. Mar Ecol Prog Ser. 2010;408:275–93.

[84] Gallucci VF, Taylor IG, Erzini K. Conservation and management of exploited shark populations based on reproductive value. Can J Fish Aquat Sci. 2006;63(4):931–42.

[85] Kinney MJ, Simpfendorfer CA. Reassessing the value of nursery areas to shark conservation and management. Conserv Lett. 2009;2(2):53–60. doi: 10.1111/j.1755-263X.2008.00046.x

[86] Ramírez-Mosqueda E, Pérez-Jiménez JC, Mendoza-Carranza M. Reproductive parameters of the southern stingray *Dasyatis americana* in southern Gulf of Mexico. Lat Am J Aquat Res. 2012;40(2):335–44. doi: 10.3856/vol40-issue2-fulltext-8

[87] Branch TA, Watson R, Fulton EA, Jennings S, McGilliard CR, Pablico GT, et al The trophic fingerprint of marine fisheries. Nature. 2010;468(7322):431–5. http://www.nature.com/nature/journal/v468/n7322/abs/nature09528.html - supplementary-information. doi: 10.1038/nature09528 21085178 10.1038/nature09528

[88] Pauly D, Christensen V, Froese R, Palomares M. Fishing Down Aquatic Food Webs Industrial fishing over the past half-century has noticeably depleted the topmost links in aquatic food chains. Am Sci. 2000;88:46–51.

[89] Brooks B. Not drowning, waving!: Safety management and occupational culture in an Australian commercial fishing port. Saf Sci. 2005;43(10):795–814. https://doi.org/10.1016/j.ssci.2005.02.007.

[90] Salminen S. Have young workers more injuries than older ones? An international literature review. J Saf Res. 2004;35(5):513–21. https://doi.org/10.1016/j.jsr.2004.08.005. 10.1016/j.jsr.2004.08.005 15530925

[91] Piniella F, Fernández-Engo MA. Towards system for the management of safety on board artisanal fishing vessels: Proposal for check-lists and their application. Saf Sci. 2009;47(2):265–76. https://doi.org/10.1016/j.ssci.2008.04.005.

[92] Le Floc’h P, Daurès F, Bihel J, Boncoeur J, Brigaudeau C, Thébaud O, editors. Analyzing fishermen behaviour face to increasing energy costs–A French case study. ICES Annual Science Conference; 2007 2007; Helsinki.

[93] Salas S, Sumaila UR, Pitcher T. Short-term decisions of small-scale fishers selecting alternative target species: a choice model. Can J Fish Aquat Sci. 2004;61(3):374–83.

[94] Peruso L, Weldon RN, Larkin SL. Predicting optimal targeting strategies in multispecies fisheries: a portfolio approach. Mar Resour Econ. 2005;20(1):24–45.

[95] Zetina-Rejón MJ, Cabrera-Neri E, López-Ibarra GA, Arcos-Huitrón NE, Christensen V. Trophic modeling of the continental shelf ecosystem outside of Tabasco, Mexico: A network and modularity analysis. Ecol Model. 2015;313:314–24. https://doi.org/10.1016/j.ecolmodel.2015.07.001.

[96] Belkin IM, Cornillon PC, Sherman K. Fronts in Large Marine Ecosystems. Prog Oceanogr. 2009;81(1–4):223–36. https://doi.org/10.1016/j.pocean.2009.04.015.

